# IL-33 contributes to sepsis-induced long-term immunosuppression by expanding the regulatory T cell population

**DOI:** 10.1038/ncomms14919

**Published:** 2017-04-04

**Authors:** Daniele C. Nascimento, Paulo H. Melo, Annie R. Piñeros, Raphael G. Ferreira, David F. Colón, Paula B. Donate, Fernanda V. Castanheira, Aline Gozzi, Paula G. Czaikoski, Wanda Niedbala, Marcos C. Borges, Dario S. Zamboni, Foo Y. Liew, Fernando Q. Cunha, Jose C. Alves-Filho

**Affiliations:** 1 Departments of Pharmacology, Ribeirao Preto Medical School, University of Sao Paulo, Ribeirao Preto 14049-900, Brazil; 2 Department of Internal Medicine, Ribeirao Preto Medical School, University of Sao Paulo, Ribeirao Preto 14049-900, Brazil; 3 Department of Immunology, Institute of Infection, Immunity and Inflammation, University of Glasgow, Glasgow G12 8TA, UK; 4 Departments of Cell Biology and Microbial Pathogenesis, Ribeirao Preto Medical School, University of Sao Paulo, Ribeirao Preto 14049-900, Brazil; 5 School of Biology and Basic Medical Sciences, Soochow University, Suzhou 215006, China

## Abstract

Patients who survive sepsis can develop long-term immune dysfunction, with expansion of the regulatory T (Treg) cell population. However, how Treg cells proliferate in these patients is not clear. Here we show that IL-33 has a major function in the induction of this immunosuppression. Mice deficient in ST2 (IL-33R) develop attenuated immunosuppression in cases that survive sepsis, whereas treatment of naive wild-type mice with IL-33 induces immunosuppression. IL-33, released during tissue injury in sepsis, activates type 2 innate lymphoid cells, which promote polarization of M2 macrophages, thereby enhancing expansion of the Treg cell population via IL-10. Moreover, sepsis-surviving patients have more Treg cells, IL-33 and IL-10 in their peripheral blood. Our study suggests that targeting IL-33 may be an effective treatment for sepsis-induced immunosuppression.

## Abstract (web summary)

Patients who survive sepsis are at increased risk of infection owing to long-term immunosuppression that is associated with an increase in Treg cell numbers. Here the authors show expansion of the Treg cell population in sepsis mice is driven by IL-33-induced ILC2 activation of IL-10 production by macrophages.

Sepsis is an systemic inflammatory response triggered by acute infection that leads to progressive multi-organ dysfunction^1^. Advances in supportive care have resulted in an increase in the survival rate of sepsis patients^2^; however, these patients have a poor long-term outcome, with risk of cognitive and physical impairments^3^,^4^,^5^.

Compelling experimental and clinical evidence indicates that sepsis can cause immunosuppression that accounts for secondary, mostly opportunistic, infections^6^,^7^,^8^,^9^. Consistent with this evidence is that patients with septic shock have an increased frequency of circulating regulatory T (Treg) cells that correlates with immunosuppression^10^,^11^. Experimentally, we and others have reported an increase in the number of Treg cells in the spleen of mice which survived sepsis (hence forward called sepsis-survivors), and involvement of these cells in long-term sepsis-induced immune dysfunction^12^,^13^. However, the mechanisms of the induction of Tregs in the long-term immunosuppression in sepsis survivors are obscure.

IL-33, an IL-1 cytokine family member, is an important mediator of type 2 immune responses^14^. Binding to a heterodimer receptor that consists of ST2 (IL-33R, IL1RL1) and IL-1 receptor accessory protein (IL-1RAcP), mature IL-33 induces the production of IL-4, IL-5, IL-10 and IL-13 from eosinophils, mast cells, T-helper 2 (Th2) cells and type 2 innate lymphoid cells (ILC2s)^15^,^16^. Moreover, IL-33 can synergize with IL-4 to promote M2 macrophage polarization^17^. Studies have also highlighted the critical role of IL-33 as an immunomodulatory cytokine that induces the expansion of Treg cell populations^18^,^19^,^20^,^21^,^22^.

Here, we show that endogenous IL-33, released in response to severe tissue damage, has an essential function in the expansion of Treg cells after sepsis and in the development of long-term sepsis-induced immunosuppression. IL-33 activates ILC2s, which produce IL-4 and IL-13 that drive M2 polarization of macrophages, resulting in the expansion of Treg cell population via the production of IL-10. Furthermore, neutralization of IL-33 with soluble ST2 (sST2, a decoy receptor for IL-33) limits the immunosuppressive effect of sepsis and reduces mortality of mice affected by secondary infection. Importantly, patients who survive sepsis have more circulating Treg cells and higher concentrations of IL-33 and IL-10 in their serum compared to healthy non-sepsis individuals. Our data, therefore, uncover a function of IL-33 in sepsis-induced immunosuppression and identify a target for potential treatment of this adverse long-term outcome induced by sepsis.

## Results

### IL-33 is critical for sepsis-induced immunosuppression

**Figure 1:**
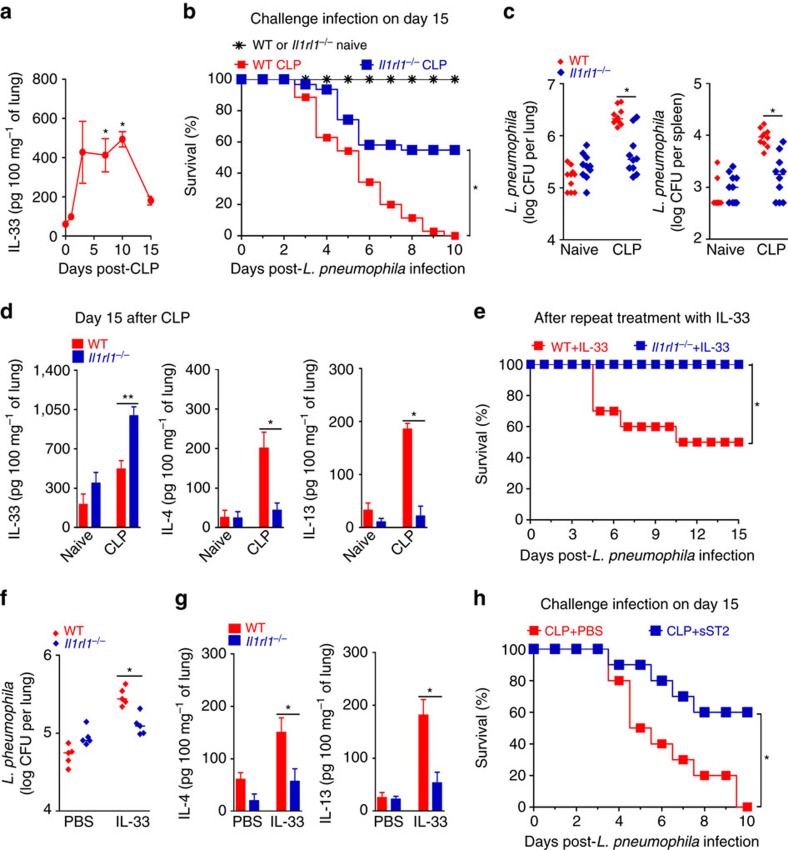
IL-33 is critical for sepsis-induced immunosuppression. (**a**) IL-33 concentrations in the lungs of BALB/c mice undergoing CLP and antibiotic treatment were determined by ELISA (*n*≥4 mice per group). (**b**–**d**) Surviving BALB/c and *Il1rl1*^*−/−*^ mice undergoing CLP and antibiotics were challenged with *L. pneumophila* 15 days after CLP. (**b**) Survival curves after challenge (*n*=30 mice for naive group, *n*=35 mice for WT CLP group and *n*=31 mice for *Il1rl1*^*−/−*^ CLP group). (**c**) Bacterial loads in lungs and spleen 24 h after challenge (*n*=10 mice per group). (**d**) Concentrations of IL-4, IL-13 and IL-33 in the lungs 15 days after CLP determined by ELISA (*n*≥4 mice per group). (**e**–**g**) BALB/c and *Il1rl1*^*−/−*^ mice were inoculated i.n. with IL-33 or PBS for 4 consecutive days and challenged i.n. 2 days later with *L. pneumophila*. (**e**) Survival curves (*n*=10 mice per group). (**f**) Bacterial load in the lung tissue 48 h after challenge (*n*=5 mice per group). (**g**) IL-4 and IL-13 concentrations in the lung tissue 48 h after challenge (*n*=5 mice per group). (**h**) BALB/c mice undergoing CLP and antibiotics were injected i.p. with sST2 or PBS. The sepsis-surviving mice were challenged with *L. pneumophila* on day 15 (*n*=10 mice per group). *P<0.05 and **P<0.01 (one-way ANOVA result with Dunnett posthoc tests in **a**, Mantel–Cox log-rank test in **b**,**e**,**h**, Mann–Whitney U test in **c**,**f** and two-tailed unpaired Student's *t*-test in **d**,**g**). Data are one experiment (**h**); or representative of two (**a**,**b**,**g**) or three (**d**,**f**) independent experiments with similar results; or pooled from two (**e**) or six (**b**) independent experiments (mean±s.e.m. in **a**,**d**,**g** and median in **c**,**f**).

Severe sepsis was performed in mice using a clinically relevant model of polymicrobial peritonitis induced by caecal ligation and puncture (CLP), which has been used extensively to investigate sepsis^12^,^13^,^23^,^24^,^25^,^26^,^27^. BALB/c mice undergoing lethal CLP were treated with antibiotics (Supplementary Fig. 1a,b). In this lethal CLP model, as oppose to the sub-lethal models, all the wild type (WT) and ST2-deficient mice (*Il1rl1*^*−/−*^) would not survive the procedure unless rescued (40%) by the treatment of antibiotics. CLP led to an early and simultaneous pro- and anti-inflammatory response, as evidenced by increased levels of IL-1β, IL-6, TNF and IL-10 in serum and lungs of mice that resolved by 72 h (Supplementary Fig. 1c,d). Moreover, a rapid development of multiple organ failures was observed after CLP, as assessed by increased serum concentrations of endocan (endothelial cell dysfunction marker), creatine kinase-MB (heart injury marker), alanine aminotransferase (liver injury marker) and blood urea nitrogen (kidney function marker) (Supplementary Fig. 1e). However, all the injury/dysfunction serum markers assessed returned to basal levels by 72 h after CLP in sepsis-surviving mice treated with antibiotics (Supplementary Fig. 1c–e). Sepsis-surviving mice show a progressive clearance of bacteraemia, which was undetectable 15 days after CLP (Supplementary Fig. 1b). Nevertheless, we observed an increasing production of type 2 cytokines (IL-4 and IL-13) and IL-33 in the lung tissue homogenates and bronchial lavage (BAL) fluids of sepsis-surviving mice at later time points, starting at day 3 after CLP and remaining elevated over the course of observation (Fig. 1a; Supplementary Fig. 2).

The increased production of IL-33 in sepsis-surviving mice at a later stage of CLP prompted us to investigate whether this cytokine plays a role in the development of long-term sepsis-induced immunosuppression. As previously described^12^, the challenge of sepsis-surviving WT mice with an intranasal sub-lethal dose of *Legionella pneumophila* at day 15 after CLP resulted in 100% mortality, whereas all naive mice survived (Fig. 1b). The high-susceptibility of sepsis-surviving mice to *L. pneumophila* infection was not accompanied by changes in the production of pro-inflammatory cytokines, such as TNF and IL-6 (Supplementary Fig. 3). Intriguingly, *Il1rl1*^*−/−*^ mice that survived from sepsis were more resistant to a subsequent challenge infection with *L. pneumophila* (Fig. 1b). Consistent with these results, sepsis-surviving *Il1rl1*^*−/−*^ mice show a significantly lower *L. pneumophila* growth in the lung and spleen than in those of the WT mice (Fig. 1c). Although sepsis-surviving *Il1rl1*^*−/−*^ mice had higher production of IL-33 than WT mice, they failed to produce more IL-4 and IL-13 in the lungs compared to the naive control mice at day 15 after CLP (Fig. 1d). The increased level of IL-33 detected may reflect the accumulation of IL-33 in the absence of ST2, while IL-4/IL-13 is downstream of IL-33/ST2 signalling. In a reverse experiment, intranasal administration of recombinant IL-33 to naive WT mice daily over a 4-days period resulted in increased susceptibility to the following challenge with *L. pneumophila* infection (Fig. 1e), and impaired bacterial clearance from the lungs in a dose-dependent manner (Fig. 1f; Supplementary Fig. 4a). Treatment with IL-33 also resulted in increased production of IL-4 and IL-13 in the lungs of WT mice (Fig. 1g; Supplementary Fig. 4b). In contrast, IL-33 had no effect on similarly treated naive *Il1rl1*^*−/−*^ mice (Fig. 1e–g). These results demonstrate the important role and specificity of IL-33 in the induction of immunosuppression associated with an increased type 2 cytokines production.

We then examined the potential therapeutic effect of neutralizing IL-33 in the surviving sepsis mice using the IL-33 decoy receptor, soluble ST2 (sST2). Treatment of WT mice with sST2 significantly protected sepsis-surviving mice from the following infection with *L. pneumophila* (Fig. 1h). There were no difference in survival between *Il1rl1*^*−/−*^ and WT mice and mice treated with sST2 and vehicle with CLP-induced sepsis receiving antibiotics (Supplementary Fig. 5a,b).

### IL-33 induces ILC2 expansion in sepsis-surviving mice

**Figure 2:**
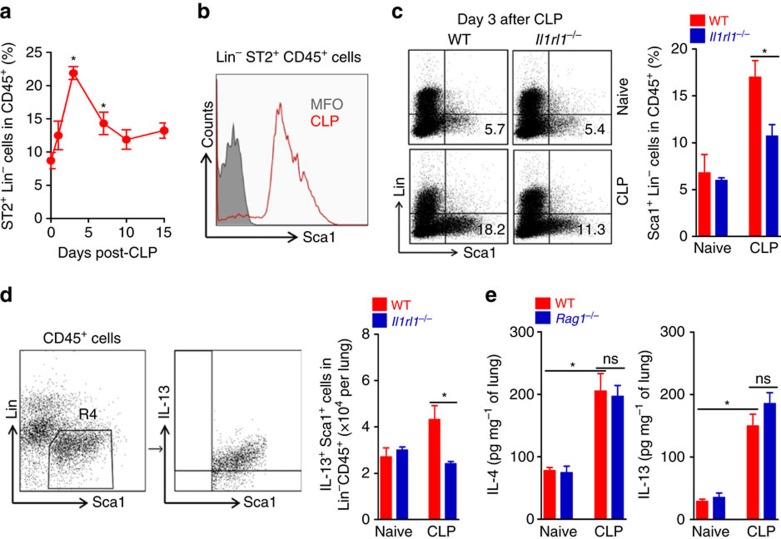
Induction of ILC2 in sepsis-surviving mice. (**a**–**e**) Lungs were harvested from BALB/c and *Il1rl1*^−/−^ mice up to 15 days after CLP. (**a**) The frequency of ILC2^+^ cells in WT mice was determined by FACS at the indicated times after CLP (*n*≥3 mice per group). (**b**) Histogram of ILC2^+^ cells (Sca1^+^ in Lin^−^CD45^+^ cells) in WT mice on day 7 after CLP. MFO, fluorescence minus one. (**c**) Representative FACS plots and frequency of ILC2^+^ cells in BALB/c and *Il1rl1*^−/−^ mice at day 3 after CLP (*n*≥3 mice per group). (**d**) Representative FACS plots and total numbers of IL-13-producing ILC2^+^ (IL-13^+^Sca1^+^Lin^−^CD45^+^) cells (*n*=5 mice per group). (**e**) IL-4 and IL-13 concentrations in the lung tissue of C57BL/6 J and *Rag1*^−/−^ mice 15 days after CLP determined by ELISA (*n*≥5 mice per group). *P<0.05 and **P<0.01 (one-way ANOVA result with Dunnett posthoc tests in **a**, two-tailed unpaired Student's *t*-test in **c**,**d**,**e**). Data are from one experiment (**d**) or representative of two (**a**–**c**,**e**) independent experiments (mean±s.e.m. in **a**,**c**–**e**).

IL-33 is a key inducer of ILC2 expansion, which is an important cellular source of type 2 cytokines^28^,^29^,^30^,^31^,^32^. We found a marked increase in lineage-negative (Lin^−^) (CD3^−^CD11c^−^CD19^−^F4/80^−^) ST2^+^Sca1^+^CD45^+^ ILC2s in the lung of sepsis-surviving mice that peaked on day 3 after CLP (Fig. 2a,b). ILC2s (Lin^−^Sca1^+^CD45^+^ cells) were present in mice of both C57BL/6 J and BALB/c backgrounds, although consistently more abundant in C57BL/6 J mice (Supplementary Fig. 6a). A similar increase of ILC2 was observed in the lung of sepsis-surviving *Rag1*^*−/−*^ mice that lack mature T- and B-lymphocytes (Supplementary Fig. 6b). However, ILC2s were significantly reduced in the lungs of sepsis-surviving *Il1rl1*^*−/−*^mice compared to WT mice (Fig. 2c). Correspondingly, we found a significantly lower number of IL-13-producing ILC2s in the lung of sepsis-surviving *Il1rl1*^*−/−*^ mice on day 7 after CLP compared to sepsis-surviving WT mice (Fig. 2d). Moreover, sepsis-surviving *Rag1*^*−/−*^ mice showed similar levels of IL-4 and IL-13 in the lungs as those found in sepsis-surviving WT mice (Fig. 2e). Altogether these results indicate that IL-33 plays a crucial role in the expansion of ILC2s after CLP-induced sepsis, which are mediating the early production of type 2 cytokines in sepsis-surviving mice.

### Type 2 cytokines mediate sepsis-induced immunosuppression

**Figure 3:**
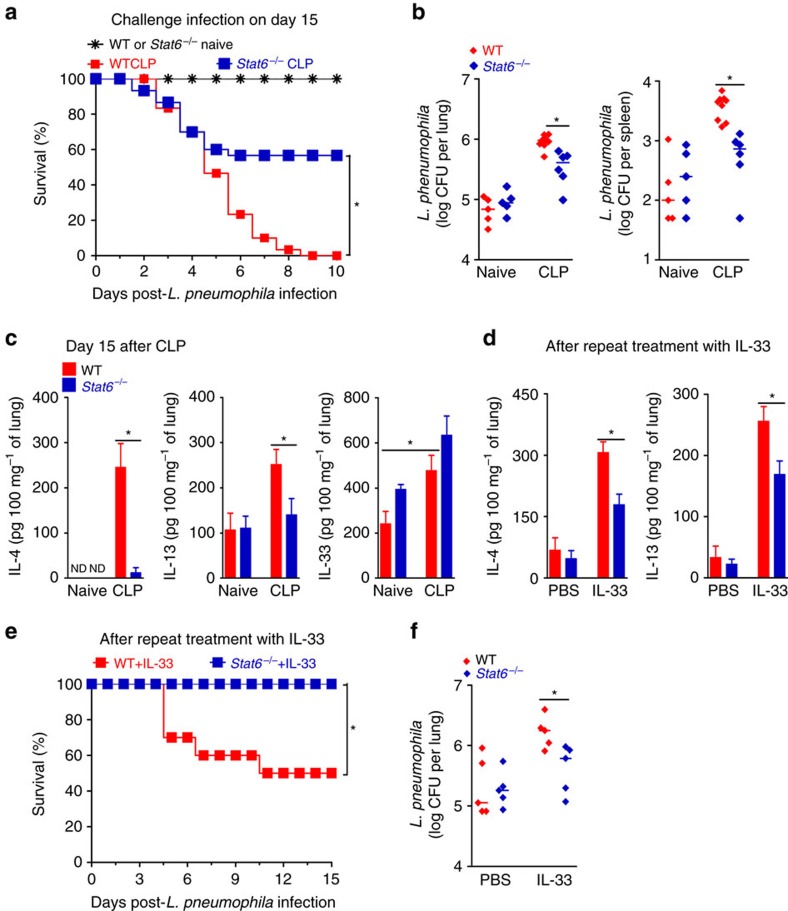
STAT6 is required for sepsis-induced immunosuppression. (**a**–**c**) Surviving BALB/c and *Stat6*^*−/−*^ mice undergoing CLP and antibiotic treatment were challenged with *L. pneumophila* or killed 15 days after CLP. (**a**) Survival curves after challenge (*n*=25 mice for naive group and *n*=30 mice for CLP group per genotype). (**b**) Bacterial load in lungs and spleen 24 h after challenge (*n*≥5 mice per group). (**c**) IL-4, IL-13 and IL-33 concentrations in the lungs after CLP determined by ELISA (*n*≥3 mice per group). (**d**–**f**) BALB/c and *Stat6*^*−/−*^ mice were treated i.n. with IL-33 (3 μg per mouse per day) or PBS for 4 consecutive days, and challenged 2 days later with *L. pneumophila*. (**d**) IL-4 and IL-13 concentrations in lung tissue 48 h after challenge (*n*≥3 mice per group). (**e**) Survival curves of mice after challenge (*n*=10 mice per genotype). (**f**) Bacterial load in the lungs 48 h after challenge (*n*=5 mice per group). *P<0.05 (Mantel–Cox log-rank test in **a**,**e**, Mann–Whitney U test in **b**,**f** and two-tailed unpaired Student's *t-*test in **c**,**d**). Data are representative of two (**d**) or three (**b**,**c**,**f**) independent experiments; or pooled from two (**e**) or five (**a**) independent experiments (median in **b**,**f** and mean±s.e.m. in **c**,**d**).

The prominent features of ILC2s and type 2 cytokines in sepsis-induced susceptibility to secondary infection prompted us to examine the role of these cytokines in the development of sepsis-induced immunosuppression. To this end, we performed experiments on *Stat6*^*−/−*^ mice, since signalling for both IL-4 and IL-13 is markedly impaired in the absence of STAT6 (ref. 33). There was no difference in survival between *Stat6*^*−/−*^ and WT mice with CLP-induced sepsis receiving antibiotics (Supplementary Fig. 5c). However, sepsis-surviving *Stat6*^*−/−*^ mice were more resistant to subsequent infection with *L. pneumophila* (Fig. 3a). Moreover, sepsis-surviving *Stat6*^*−/−*^ mice showed significantly lower bacterial burdens in the lungs and spleen than those of the WT mice after *L. pneumophila* challenge (Fig. 3b). Similar to findings obtained with sepsis-surviving *Il1rl1*^*−/−*^ mice, the production of IL-4 and IL-13 was markedly reduced in the lungs of *Stat6*^*−/−*^ mice on day 15 after CLP (Fig. 3c), indicating that these type 2 cytokines can induce their synthesis in a positive feedback loop in an STAT6-dependent manner. However, STAT6-deficiency did not reduce the amount of IL-33 in the lungs of sepsis-surviving mice (Fig. 3c), again indicating that the function of IL-33 is upstream of type 2 cytokines in the present system. Indeed, repeated intranasal administration of IL-33-induced lower levels of IL-4 and IL-13 production in the lungs of *Stat6*^*−/−*^ mice compared to that of the WT mice (Fig. 3d). Moreover, exogenous IL-33 treatment did not affect the resistance of *Stat6*^*−/−*^ mice to subsequent challenge infection with *L. pneumophila* (Fig. 3e,f). Collectively, these results indicate that type 2 cytokines mediate the development of sepsis-induced immunosuppression, an effect regulated by IL-33/ST2 signalling.

### Polarization of M2-like macrophages in sepsis-surviving mice

**Figure 4:**
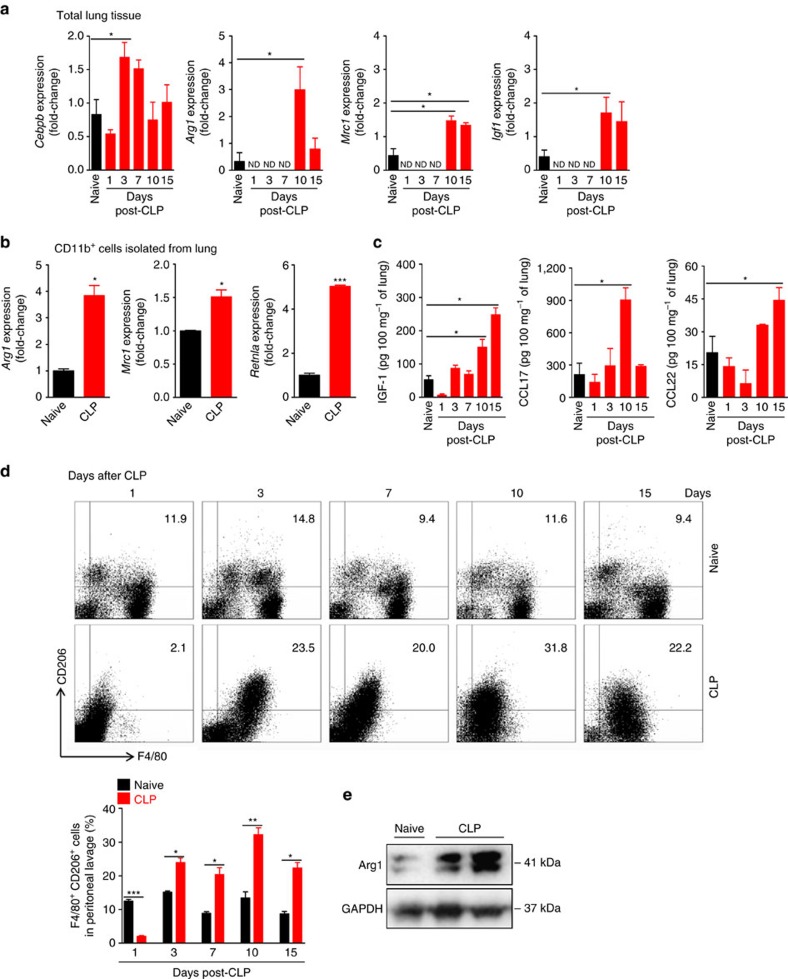
Sepsis induces polarization of M2-like macrophages. (**a**–**e**) Lung tissue and peritoneal cells from C57BL/6 J (**a**,**c**,**d**) and BALB/c (**b**,**e**) mice undergoing CLP and antibiotic treatment were harvested at the indicated time points. (**a**) mRNA expression of *Cebpb* (C/EBPβ)*, Arg1* (arginase-1), *Mrc1* (MR) *and Igf1* in the total lungs were determined by RT-qPCR at indicated times after CLP (*n*≥4 mice per group). (**b**) Lungs obtained from six mice, either naive or CLP (day 10 after CLP), were pooled from two independent samples and CD11b^+^ cells were isolated. mRNA expression of *Arg1* (arginase-1), *Mrc1* (MR), *Rentla* (encoding Fizz1) in isolated CD11b^+^ cells were determined by RT-qPCR. (**c**) IGF-1, CCL17 and CCL22 concentrations in the lungs were determined by ELISA at indicated times after CLP (*n*≥3 mice per group). (**d**) Representative FACS plots and frequency of peritoneal CD206^+^F4/80^+^ macrophages at indicated times after CLP (*n*≥3 mice per group). (**e**) Representative western blot of Arg1 (Arginase-1) expression in the peritoneal cells at day 10 after CLP (*n*=9 for naive group and *n*=5 for CLP group). ND, not detected. *P<0.05, **P<0.01 and ***P<0.001 (one-way ANOVA result with Dunnett posthoc tests in **a**,**c**, two-tailed unpaired Student's *t*-test in **b**,**d**). Data are from one (**b**) and representative of two (**a**,**c**–**e**) independent experiments (mean±s.e.m. in **a**–**d**).

IL-4 and IL-13 are prototypical cytokines that drive the polarization of macrophage toward an M2 phenotype^34^. We next investigated the kinetics of macrophage polarization in mice after sepsis induction. We found up-regulation of *Cebpb* mRNA, a transcription factor required for expression of M2-associated genes^35^, in the lung tissue of mice by day 3 and 7 after CLP (Fig. 4a). Moreover, we also detected high-expression of the canonical M2-associated transcripts, *Arg1* (encoding arginase-1) and *Mrc1* (encoding mannose receptor, CD206), as well as an up-regulation of the wound healing-related gene *Igf1* (encoding IGF-1), in the lung tissues of sepsis-surviving mice by day 10 and 15 after CLP (Fig. 4a). Concordantly, increased expression of *Arg1, Mrc1 and Retnla* (encoding Fizz-1) was found in a CD11b^+^ macrophage-enriched population isolated from the lungs of sepsis-surviving mice by day 10 after CLP (Fig. 4b). Consistent with these results, high concentrations of IGF-1 and M2-derived chemokines CCL17 and CCL22 protein were found in the lung tissue homogenates by day 10 and 15 after CLP (Fig. 4c). Furthermore, we also found increased expression of CD206 in F4/80^low^peritoneal macrophages from sepsis-surviving mice by day 3 after CLP; reaching peak levels at day 10 and maintained at an elevated level by day 15 (Fig. 4d). Consistent with this observation, increased expression of Arg1 protein was also observed in peritoneal macrophages harvested from sepsis-surviving mice 15 days after CLP when compared to that of the naive mice (Fig. 4e). Low-expression of F4/80 is characteristic of macrophages recruited to the serous cavities under inflammatory conditions^36^,^37^.

**Figure 5:**
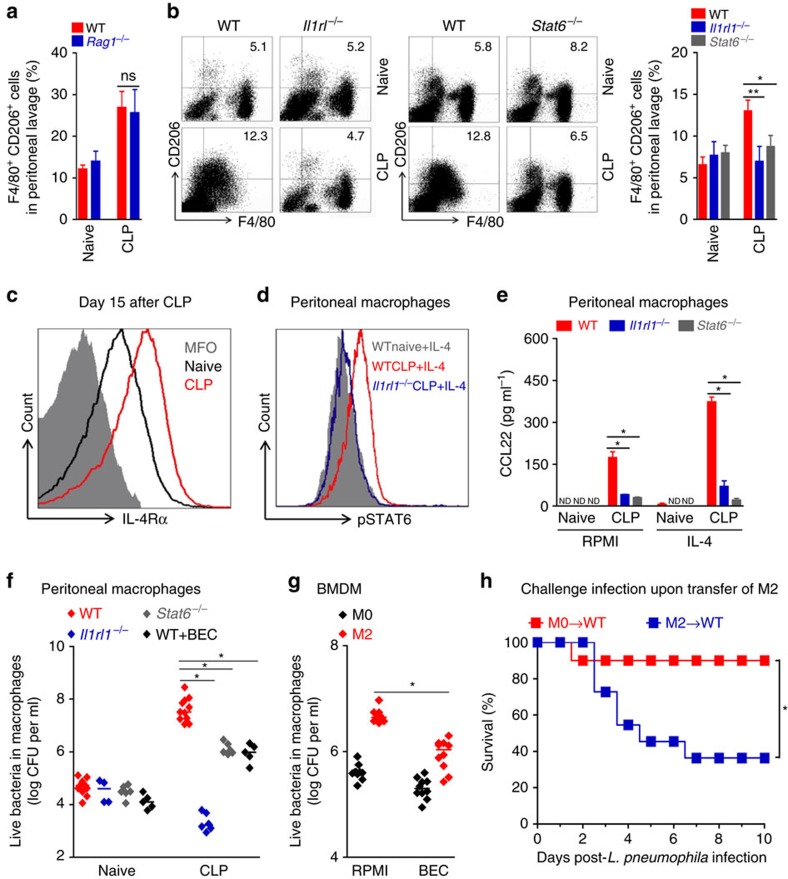
IL-33 and STAT6 promote M2 differentiation after sepsis. (**a**) Peritoneal cells were collected from C57BL/6 J or *Rag1*^−/−^ mice 15 days after CLP and antibiotic treatment and the frequency of F4/80^+^CD206^+^ macrophages determined by FACS (*n*≥3 mice per group). (**b**) Peritoneal cells of BALB/c, *Il1rl1*^−/−^ and *Stat6*^*−/−*^ mice were harvested in ‘**a**' above and the frequency of F4/80^+^CD206^+^ macrophages determined by FACS (*n*≥5 mice per group). (**c**) Representative FACS plots of IL-4Rα^+^F4/80^+^ peritoneal macrophages of C57BL/6 J mice at day 15 after CLP (*n*=5 per group). (**d**,**e**) Peritoneal macrophages harvested in **b** above were stimulated with IL-4 *in vitro*. (**d**) STAT6 phosphorylation in the cells was determined by FACS (*n*=4 per group). (**e**) Concentrations of CCL22 in the 18 h culture supernatants determined by ELISA (*n*=4 mice per group). (**f**) Peritoneal cells of BALB/c, *Il1rl1*^−/−^ and *Stat6*^*−/−*^ mice were harvested in **a** above. The number of viable bacteria recovered from lysates of peritoneal macrophages exposed, *in vitro*, to *L. pneumophila* for 72 h in the presence or absence of arginase inhibitor BEC. (**g**) BALB/c macrophages from BMDM were cultured for 2 days with M-CSF (M0) or IL-4, IL-13 and IL-33 (M2). The number of viable bacteria recovered from lysates of M2-polarized macrophages exposed, *in vitro*, to *L. pneumophila* for 72 h in the presence or absence of BEC. (**h**) M0 or M2 macrophages were adoptively transferred (4 × 10^6^ cells, i.v.) into naive BALB/c mice, which were challenged 12 days later with *L. pneumophila*. Survival of mice was recorded (*n*=10 mice for M0 group and *n*=11 for M2 group). ND, not detected. *P<0.05, **P<0.01 and ***P<0.001 (two-tailed unpaired Student's *t-*test in **a**,**b**, one-way ANOVA result with Bonferroni's posthoc tests in **d**, Mantel-Cox log-rank test in **e**). Data are representative of two (**a**,**c**–**g**) independent experiments; or pooled from two (**b**,**h**) experiments (mean±s.e.m. in **a**,**b**,**e**–**h**).

As demonstrated previously^17^, we confirmed that IL-33 strongly enhances the polarization of IL-4 treated bone marrow-derived macrophages (BMDMs) toward M2 phenotype, as assessed by the CD206 expression (by flow cytometry) and production of CCL22 (by ELISA), an effect that was abrogated in *Il1rl1*^*−/−*^ BMDMs (Supplementary Fig. 7). Moreover, the expression of M2 markers was also completely absent in IL-4 treated *Stat6*^*−/−*^ BMDMs (Supplementary Fig. 7). We then addressed the contribution of type 2 cytokines and IL-33 on M2 polarization of macrophages in sepsis-surviving mice. Consistent with the increased production of IL-4 and IL-13 found in the sepsis-surviving *Rag1*^*−/−*^ mice (Fig. 2e), adaptive immunity was not required for the polarization of peritoneal macrophages toward M2 phenotype, since sepsis-surviving *Rag1*^*−/−*^ mice showed a similar frequency of peritoneal macrophage expressing CD206 as the WT mice (Fig. 5a). Instead, we found that ST2 and STAT6 signalling were required for M2 polarization since the expression of CD206 was not increased in peritoneal macrophages from sepsis-surviving *Il1rl1*^*−/−*^ or *Stat6*^*−/−*^ mice (Fig. 5b). Accordingly, arginase activity assessed indirectly by urea production was considerably lower in the peritoneal lavage and BAL fluids from sepsis-surviving *Il1rl1*^*−/−*^ and *Stat6*^*−/−*^ mice than in those from the WT controls (Supplementary Fig. 8a,b). Moreover, production of IGF-1 protein in the lung was also reduced in the lungs of sepsis-surviving *Il1rl1*^*−/−*^ or *Stat6*^*−/−*^ mice (Supplementary Fig. 8c).

Previous studies demonstrated that IL-33 signalling amplifies M2 macrophage polarization through up-regulation of IL-4 receptor-α (IL-4Rα) expression, resulting in an increased IL-4 response^17^. We then analysed peritoneal macrophages from sepsis-surviving mice by flow cytometry and observed increased expression of IL-4Rα compared to that of the naive mice (Fig. 5c). Accordingly, IL-4 stimulation induced higher STAT6 phosphorylation in peritoneal macrophages from sepsis-surviving WT mice than that observed in the macrophages from naive mice, an effect that was abrogated in macrophages from sepsis-surviving *Il1rl1*^*−/−*^ mice (Fig. 5d). Consistent with these findings, peritoneal macrophages from sepsis-surviving WT mice released a larger amount of CCL22 in the presence of IL-4, while macrophages from *Il1rl1*^*−/−*^ and *Stat6*^*−/−*^ mice produced a low-level of this M2-defived chemokine (Fig. 5e).

While M2 macrophages are involved in tissue repair and immune-regulation, bactericidal activity is considered a cardinal feature of M1 macrophages^38^. We next investigated the bactericidal activity of M2-like macrophages from surviving sepsis mice. To this end, peritoneal macrophages isolated from sepsis-surviving mice at day 15 after CLP were incubated with *L. pneumophila* and intracellular bactericidal activity was assessed by quantification of the intracellular viable bacterial load. Macrophages from sepsis-surviving mice showed substantially more intracellular bacterial load than macrophages from the control naive mice indicating impaired bactericidal ability of the macrophages from sepsis-surviving mice. Importantly, macrophages recovered from sepsis-surviving *Il1rl1*^*−/−*^ and *Stat6*^*−/−*^ mice harboured significantly fewer bacteria than macrophages from sepsis-surviving WT mice (Fig. 5f), showing the key role of ST2 and STAT6 in the impaired bactericidal activity of the macrophage from sepsis-surviving mice. Interestingly, pharmacological inhibition of arginase with S-(2-boronoethyl)-l-cysteine (BEC) markedly enhanced the bactericidal activity of macrophages from sepsis-surviving mice, indicating that arginase activity contributes, at least in part, to the impaired bactericidal ability of these cells (Fig. 5f). Consistent with these findings, BMDMs polarized to M2 phenotype also exhibited reduced ability to control intracellular *L. pneumophila* growth compared with non-polarized M0 macrophages. The bactericidal activity of the polarized M2 macrophages was also improved by arginase inhibition (Fig. 5g).

We then determined the role of M2 macrophages in the susceptibility of mice to *L. pneumophila* infection *in vivo*. Adoptive transfer of M2-polarized BMDMs, but not M0, into naive mice increased the susceptibility of the recipients to a challenge with *L. pneumophila* infection (Fig. 5h), similar to those observed with exogenous IL-33 treatment (Fig. 1e). Taken together, these data demonstrate a robust macrophage reprogramming toward an M2 phenotype in sepsis-surviving mice induced by type 2 cytokines and IL-33, presumably associated with the development of a wound healing response. However, the switch of macrophages toward M2 polarization may contribute to the development of long-term sepsis-induced immunosuppression.

### M2 promotes Treg differentiation in sepsis-surviving mice

**Figure 6:**
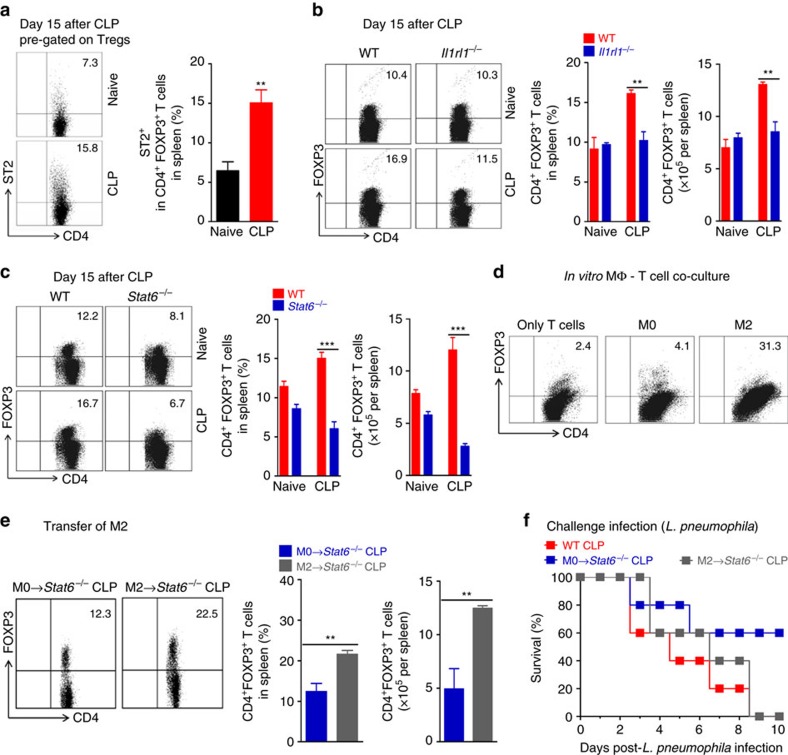
IL-33 and STAT6 increase Treg cell population after sepsis. (**a**–**c**) Spleen cells from BALB/c, *Il1rl1*^*−/−*^ or *Stat6*^*−/−*^ mice undergoing CLP and antibiotic treatment were collected 15 days after CLP. Frequency and number of ST2^+^Foxp3^+^CD4^+^ and Foxp3^+^CD4^+^ lymphocytes were analysed by FACS (*n*≥4 mice per group). (**d**) C57BL/6 J macrophages from BMDM were cultured for 2 days with M-CSF (M0) or IL-4, IL-13 and IL-33 (M2). Naive CD4^+^ T cells (CD4^+^CD25^−^ T cells) were then cultured for 4 days with M0 or M2 in the presence of IL-2 and anti-CD3 antibody. Percentage of CD4^+^Foxp3^+^ lymphocytes was analysed by FACS (pooled from five mice and pooled from five well per group). (**e**,**f**) M0 or M2 macrophages were adoptively transferred (4 × 10^6^ cells, i.v., on day 3 after CLP) into *Stat6*^*−/−*^ sepsis-surviving mice. The surviving mice were either killed or challenged with *L. pneumophila* on day 15 after CLP. (**e**) Representative FACS plots, frequency and number of Foxp3^+^ CD4^+^ T cells (*n*=3 mice per group). (**f**) Survival curves of mice after *L. pneumophila* challenge (*n*=5 mice per group). *P<0.05, **P<0.01 and ***P<0.001 (two-tailed unpaired Student's *t*-test in **a**–**c**,**e**, Mantel–Cox log-rank test in **f**). Data are representative of one (**f**), two (**a**,**e**) or four (**b**,**d**) independent experiments (mean±s.e.m. in **a**–**c**,**e**).

Since IL-33 can expand Treg cell population and these cells have been shown to mediate sepsis-induced immunosuppression, we next investigated the potential function of IL-33 in the induction of Treg cells in CLP-induced immune dysfunction. At day 15 after CLP, a significantly higher frequency and an absolute number of CD4^+^Foxp3^+^ Treg cells expressing ST2 were found in the spleen of sepsis-surviving mice as compared to naive mice (Fig. 6a,b). However, the frequency and the absolute number of Treg cells were not increased in the spleens of sepsis-surviving *Il1rl1*^*−/−*^ or *Stat6*^*−/−*^ mice (Fig. 6b,c). Since ST2 and STAT6 signalling were critical to induce M2 polarization in sepsis-surviving mice, we examined whether M2 macrophages might induce the generation of Treg cells. BMDMs from naive mice (M0) were polarized into M2 (with IL-33/IL-4/IL-13) and co-cultured with naive CD25^−^CD4^+^ T cells in the presence of exogenous IL-2 and anti-CD3 but in the absence of TGF-β. As expected, few naive CD4^+^ T cells expressed Foxp3 after 4 days under this culture condition. However, Μ2 macrophages, but not M0, substantially increased the percentage of CD4^+^ T cell expressing Foxp3 (Fig. 6d). We then examined whether M2 macrophages were responsible for the expansion of Treg cells in sepsis-surviving mice. M2 macrophages were adoptively transferred into *Stat6*^*−/−*^ mice 3 days after CLP induction and Treg cell generation was monitored. After 12 days, M2-recipient *Stat6*^*−/−*^ mice showed a marked increase in the frequency and the total number of Treg cells in their spleen compared to those receiving M0 macrophages or PBS (Fig. 6e; Supplementary Fig. 9a). These mice also had impaired resistance to a subsequent challenge infection with *L. pneumophila* (Fig. 6f; Supplementary Fig. 9b). Collectively, these data indicate that ST2 signalling and M2 macrophages are involved in the expansion of Treg cell population in sepsis-surviving mice.

### IL-10 promotes Treg cell expansion in sepsis-surviving mice

**Figure 7:**
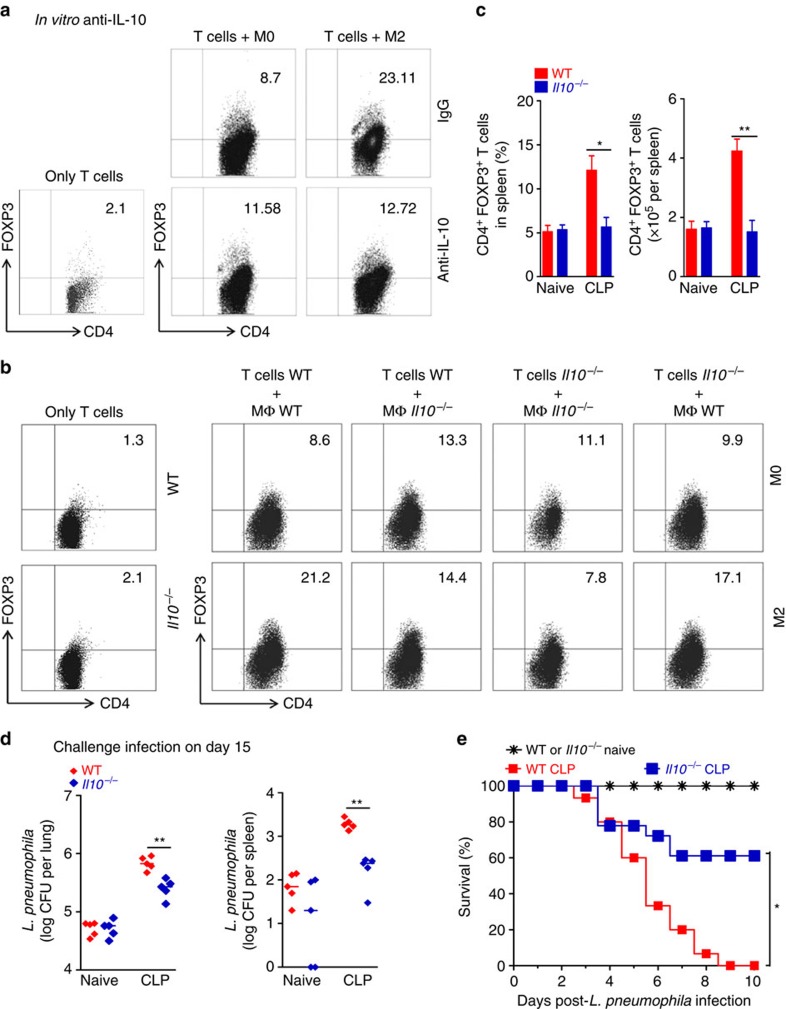
Expansion of Treg cell population by M2 is IL-10-dependent. (**a**,**b**) BMDM from WT or *Il10*^*−/−*^ mice were polarized to M0 or M2 phenotype and co-cultured for 4 days with CD4^+^CD25^−^ T cells (purified from spleen of C57BL/6 J or *Il10*^*−/−*^ naive mice) in the presence of IL-2, anti-CD3 and anti-IL-10, or medium alone. Percentage of CD4^+^Foxp3^+^ Treg cells was analysed by FACS. (both cell types pooled from five mice and pooled from five well per group). (**c**–**e**) Sepsis-surviving C57BL/6 J and *Il10*^*−/−*^ mice were either killed or challenged with *L. pneumophila* on day 15 after CLP. (**c**) Frequency and the total number of Foxp3^+^CD4^+^ T cells in the lungs (*n*≥3 mice per group). (**d**) Bacterial load in the lungs and spleen 24 h after challenge (*n*=5 mice per group). (**e**) Survival curves of mice after challenge (*n*=15 mice for naive group, *n*=15 mice for WT CLP group and *n*=18 mice for *Il10*^*−/−*^ CLP group). *P<0.05, **P<0.01 and ***P<0.001 (two-tailed unpaired Student's *t*-test in **c**, Mann–Whitney U test in **d**, Mantel–Cox log-rank test in **e**). Data are representative of three (**c**,**d**) independent experiments or pooled of three (**e**) independent experiments (mean±s.e.m. in **c**, median in **d**).

To further address the mechanism underlining the enhancement of Treg differentiation by M2 macrophages, we investigated the contribution of IL-10. Neutralization of IL-10 bioactivity with anti-IL-10 antibody markedly reduced the expression of Foxp3 in CD4^+^ T cells induced by co-culture with M2 macrophages (Fig. 7a). Moreover, *Il10*^*−/−*^ M2 macrophages induced lower frequency of CD4^+^Foxp3^+^ T cells than WT M2 macrophages when co-cultured with CD4^+^CD25^−^ T cells from WT or *Il10*^*−/−*^ mice (Fig. 7b). These data suggest that M2 macrophage-derived IL-10 is involved in the expansion of Treg cells in sepsis-surviving mice.

We then examined whether IL-10 participates in the pathogenesis of immune dysfunction in sepsis-surviving mice. In agreement with data from M2 polarization kinetics, we found an increased concentration of IL-10 in the lungs of sepsis-surviving mice by day 3 after CLP, remaining elevated over the course of observation (Supplementary Fig. 10a). Concordantly, sepsis-surviving *Rag1*^*−/−*^ mice, which had increased the polarization of M2 macrophages (Fig. 5a), also showed elevated concentration of IL-10 in the lungs (Supplementary Fig. 10b). In contrast, the production of IL-10 in the lungs of *Il1rl1*^*−/−*^ and *Stat6*^*−/−*^ mice was markedly reduced at day 15 after CLP (Supplementary Fig. 10c,d), suggesting that type 2 cytokines and IL-33 are involved in the production of IL-10 in sepsis-surviving mice. Interestingly, we found that *Il10*^*−/−*^ and WT mice show a high and comparable concentration of IL-4 and IL-33 in the lungs and a similar frequency of peritoneal M2 macrophages (F4/80^+^CD206^+^ cells) at day 15 after CLP (Supplementary Fig. 11). Thus, these data suggest that IL-10 is not required for polarization of macrophage toward M2-like phenotype and acting downstream of M2 macrophages in sepsis-surviving mice. The frequency and the absolute number of splenic CD4^+^Foxp3^+^ Treg cells were marked reduced in *Il10*^*−/−*^ sepsis-surviving mice at day 15 after CLP (Fig. 7c). Finally, sepsis-surviving *Il10*^*−/−*^ mice show significantly lower bacterial burdens in the lungs and spleen and higher survival rate to challenge with *L. pneumophila* infection than the WT control mice (Fig. 7d,e). There were no differences in survival between *Il10*^*−/−*^ and WT mice with CLP-induced sepsis receiving antibiotics (Supplementary Fig. 5d). Altogether, these data indicate that IL-10 derived from M2 macrophages is required for Treg cells expansion and, consequently, for the immune dysfunction in sepsis-surviving mice.

### Sepsis survivors have more Treg cells, IL-33 and IL-10

**Figure 8:**
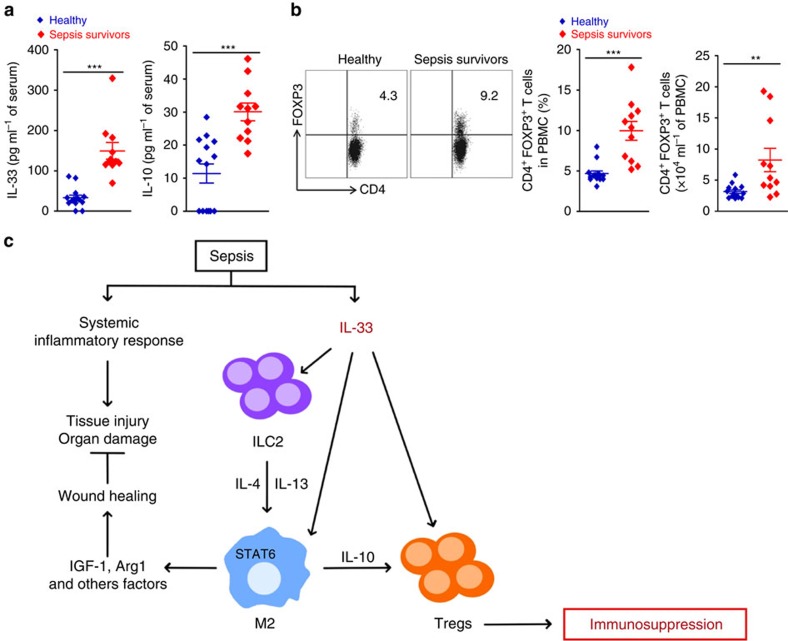
IL-33 and Treg cell population in sepsis-surviving patients. (**a**,**b**) Peripheral blood from sepsis-surviving patients and healthy controls (*n*=11 patients*, n*=14 healthy) was examined for (**a**) concentrations of IL-33 and IL-10 by ELISA and (**b**) frequency and number of CD4^+^Foxp3^+^ T cells by FACS. **P<0.01 and ***P<0.001 (two-tailed unpaired Student's *t*-test in **a**,**b**). Data are mean±s.e.m. in **a**,**b**. (**c**) IL-33, released during the widespread tissue injury, induces ILC2s expansion and M2 macrophages in sepsis survivors, promoting a wound healing process. However, IL-33-induced IL-10-secreting M2 macrophages increase the expansion of Treg cells, thereby contributing to the development of long-term sepsis-induced immunosuppression.

To demonstrate the clinical relevance of our findings, we examined sepsis-surviving patients. A total of 11 patients admitted to the intensive care unit, who met the inclusion and exclusion criteria and survived after treatment of sepsis were prospectively enroled into the study. Peripheral blood from sepsis survivors was collected 5–10 months after sepsis diagnosis and analysed for the frequency, and the absolute number of CD4^+^Foxp3^+^ T cells and serum concentration of IL-33 and IL-10 together with age- and sex-matched healthy volunteer blood donors (*n*=14). The demographic and clinical characteristics of the patients are shown in Supplementary Tables 1 and 2. The total number of leukocytes was increased in sepsis survivors when compared to healthy controls, which was mainly due to the increase in the number of monocytes (Supplementary Fig. 12). Sepsis-surviving patients had significantly higher concentrations of IL-33 and IL-10 in their serum and more circulating Treg cells compared to those of the healthy controls (Fig. 8a,b). These data are consistent with the notion that our finding in the mouse model (summarized in Fig. 8c) may be translated to the clinical scenario.

## Discussion

Data presented here show that IL-33/ST2 signalling leads to a significant expansion of Foxp3^+^ Treg cell population expressing ST2 in sepsis-surviving mice. This mechanism not only accounts for, at least in part, the development of long-term sepsis-induced immune dysfunction but also suggests that blocking IL-33 or ST2 are potential targets for treating this immunological sequel in sepsis survivors.

Epithelial and endothelial cells in most organs express IL-33 constitutively^39^, which can act as an endogenous danger signal or alarmin released in response to tissue damage^40^. Moreover, myeloid cells can also release IL-33 in response to pro-inflammatory cytokines or TLR ligands^41^,^42^. In this study, we show that IL-33 increases in response to the systemic inflammation and tissue damage caused by experimental septic peritonitis, remaining elevated in the lung tissue for days after recovery from sepsis. Moreover, we show that IL-33 induces the expansion of ILC2 populations and the subsequent production of IL-4 and IL13, which together with IL-33 induce a pronounced macrophage reprogramming toward an M2-like phenotype in sepsis-surviving mice. In the absence of ST2, ILC2 expansion and type 2 cytokines production is abolished. Also, increased production of IL-4 and IL-13, as well as M2 polarization, is maintained in sepsis-surviving *Rag1*^*−/−*^mice. These results suggest that IL-33-induced ILC2s rather than T helper-2 cells are likely the source of type 2 cytokines observed in sepsis survivors, but the participation of other cells types such as macrophages is not excluded. Conversely, sepsis-surviving mice lacking ST2 or STAT6 do not show polarization of M2-like macrophage, indicating that type 2 cytokines and IL-33 are critically involved in the polarization of M2 macrophages in sepsis-surviving mice. Consistent with this observation, increased number of circulating M2 macrophage was reported in septic patients^43^.

In the CLP-induced sepsis, there is no complete resolution of the inflammation as long as the abscesses surrounding the devitalized caecum remains in survivors. Indeed, it was previously reported that IL-13 concentration increases in the lungs and kidneys of mice during late CLP and that its neutralization worsens tissue inflammation and organ dysfunction^44^. Consistent with the effect of IL-13 on M2 polarization, we found that many factors involved in tissue repair, including Arg1, IGF-1 and TGF-β1 (ref. 45), are up-regulated in lungs of sepsis-surviving mice. In line with this observation, both ILC2s and M2 macrophages have been reported to be involved in the wound healing process and tissue repair^30^,^45^. Collectively, our results illustrate the dynamic series of molecular and cellular events in CLP-induced sepsis survivors, in which IL-33 may primarily function as an alarmin that rapidly responds to injurious signals and provides early instruction toward the repair process.

Earlier studies have demonstrated an increase in the number of Treg cells in the spleen of sepsis-surviving mice, which are involved the development of long-term sepsis-induced immune dysfunction^12^,^13^. Herein we show that genetic deletion of ST2 and STAT6 prevents the expansion of Foxp3^+^ Treg cell population in sepsis-surviving mice, concomitant with a marked improvement in the survival rate after secondary pneumonia induced by *L. pneumophila* infection. These results implicate IL-33 and IL-4/IL-13 signalling in the expansion of Treg cell population and the development of long-term sepsis-induced dysfunction. In a previous report, we showed that exogenous IL-33 can also protect mice against CLP by rapidly mobilizing neutrophils into the infectious focus to kill the pathogens during acute sepsis^46^. Thus, the induction and release of IL-33 during sepsis is a ‘trade-off' between the protection from an initial acute condition (such as resistance to primary infection and induction of tissue repair) and the subsequent manifestation of long-term immunosuppression. This ‘trade-off' could also explain why blocking IL-33/ST2 signalling could not completely protect the sepsis-surviving mice from the secondary challenge infection.

M2 macrophages can induce the differentiation of regulatory T cells^47^. Moreover, CCR4, the receptor for M2-derived chemokine's CCL17 and CCL22, plays a detrimental role in acute sepsis and contributes to the suppressive function of Tregs in sepsis-surviving mice^48^,^49^. Since ST2 and STAT6 signalling were required for the differentiation of M2 macrophages in sepsis survivors, we hypothesized that M2 macrophages might contribute to the expansion Treg cell population in sepsis-surviving mice. *In vitro*, we found that M2-polarized macrophages can efficiently induce Treg cell generation, at least in part, in an IL-10-dependent manner. Moreover, adoptive transference of M2-polarized macrophages into sepsis-surviving mice lacking STAT6 markedly induces expansion of Treg cell population and increases susceptibility to secondary pneumonia induced by *L. pneumophila*. Finally, we demonstrated that deficiency of IL-10 prevents the increase of Treg cell population in sepsis-surviving mice and improves survival from secondary pneumonia caused by *L. pneumophila*. Indeed, IL-10 can potentiate differentiation of TGFβ-induced Foxp3^+^ Treg cells via STAT3 activation^50^. Moreover, it was previously shown that neutralization of IL-4, IL-10 and TGF-β reduces expansion Treg cell population and improves cell-mediated immunity in septic mice^11^,^51^. Collectively, these data indicate that IL-33/ST2 and IL-4/IL-13/STAT6 signalling are required for the expansion of Treg cells in sepsis-surviving mice, linking IL-10-secreting M2 macrophage in this process. The Treg cells from sepsis-surviving mice expressed a significant level of ST2. It is thus possible that IL-33 could also directly activate the Treg cell populations expanded by the M2/IL-10 pathway (Fig. 8c).

Although there are differences among the subsets of macrophages such as M2 macrophage, tumour-associated macrophage, regulatory macrophage and monocytic myeloid-derived suppressor cell (MDSC), they all share several surface markers and also exhibit immune suppressive activities. In this context, it was demonstrated that IL-33 could also expand MDSCs, which in turn generate periphery-induced Foxp3^+^ Treg cells mediating cardiac and skin allograft survival^18^,^52^. Moreover, it was reported that an MyD88-dependent MDSC population that express IL-10 expanded after CLP in mice, contributing to the T cell suppression seen after sepsis^53^. Therefore, we cannot exclude the possibility that IL-33-induced MDSCs also participate in the production of IL-10 and expansion of Treg cells in sepsis survivors.

Our finding in the mouse model is likely to apply to clinical sepsis. At 5-10 months after sepsis diagnosis, sepsis survivors have significantly more circulating Foxp3^+^ Treg cells and also higher concentrations of IL-33 and IL-10 in their serum compared to those of the healthy controls. However, we have only been able to enrol into the study patients with severe sepsis or septic shock. Thus, whether the severity of septic episode would impact on the degree of the long-term immune dysfunction in sepsis survivors is currently unclear and merits further investigation together with the identification of the cellular sources of IL-33 in sepsis.

In summary, our results have identified a previously unrecognized dual role of IL-33 in sepsis survivors. IL-33, released during the widespread tissue injury, induces ILC2s expansion and M2 polarization of macrophages in sepsis survivors, promoting a wound healing process. However, IL-33-induced IL-10-secreting M2 macrophages increase the expansion of Treg cell population, thereby contributing to the development of long-term sepsis-induced immunosuppression. Our study raises the possibility of targeting IL-33 to ameliorate sepsis-induced long-term immunosuppression following recovery from infection.

## Methods

### Mice

BALB/c and C57BL/6 J mice were purchased from Charles River. *Il1rl1*^*−/−*^ (deficient for ST2) mice on C57BL/6 background were originally provided by Dr Andrew McKenzie (LMB, Cambridge)^54^. *Il1rl1*^*−/−*^ mice on BALB/c background were generated as described^55^. *Stat6*^*−/−*^ mice on BALB/c background and *Il10*^*−/−*^ and *Rag1*^*−/−*^ on C57BL/6 J background were purchased from Jackson Laboratories. All mice were bred and maintained under specific pathogen-free conditions at the Animal Facility of the School of Medicine of Ribeirão Preto, University of São Paulo. All experiments were carried out with 8-week-old male mice according to the guidelines of the Animal Welfare Committee of the School of Medicine of Ribeirão Preto, University of São Paulo (protocol number: 120/2011).

### Patients

Eleven adult patients admitted to the Emergency Department of the School of Medicine of Ribeirão Preto with severe sepsis or septic shock between October 2014 and November 2015, who met the inclusion/exclusion criteria and survived after treatment of sepsis, were prospectively enroled into the study. In addition, 14 healthy volunteers were also included in the study (Supplementary Table 1). All patients enroled fulfilled the criteria defined by the 2001 International Sepsis Definitions Conference^56^. The exclusion criteria included active haematological malignancy or cancer, chronic treatment with steroids, transplantation, HIV infection or advanced cirrhosis. Peripheral blood samples were collected from all patients 5–10 months after sepsis diagnosis. Informed written consent from all participants was obtained. The study was approved by the Human Subjects Institutional Committee of the Ribeirão Preto Medical School, Brazil (Licence number: 30459114.6.0000.5440).

### Caecal ligation and puncture model

Caecal ligation and puncture Model (CLP) was performed as previously described^12^. Briefly, mice were anaesthetized by intraperitoneal (i.p.) administration of 200 μl ketamine and xylazine diluted in phosphate-buffered saline (PBS) (100 mg kg^−1^ ketamine, 10 mg kg^−1^ xylazine). A double puncture was made through the caecum with an 18-gauge needle to induce a severe CLP sepsis. In experiments performed on BALB/c background, mice received an i.p. injection of ertapenem sodium (30 mg kg^−1^, Merck Research Laboratory) beginning 6 h after CLP and then every 12 h up to day 4. In experiments performed on the C57BL/6 J background mice, antibiotic were injected i.p. up to day 3 after CLP.

### *Legionella pneumophila* infection

Sepsis-surviving mice were infected with a virulent clinical strain of *L. pneumophila* (serogroup 1, CR1326) as described previously^57^. *L. pneumophila* were grown for 48 h on charcoal yeast extract agar (1% yeast extract, 1% *N*-(2-acetamido)-2- aminoethanesulfonic acid, pH 6.9, 3.3 mM L-cysteine, 0.33 mM Fe(NO_3_), 1.5% bacto agar and 0.2% activated charcoal). Mice were inoculation intranasally with 40 μl of a suspension containing 7 × 10^7^ *L. pneumophila*.

### Bacterial counts

Measurement of *L. pneumophila* in lung and spleen was determined as previously described^12^. Briefly, 24 h after infection, lungs and spleen were explanted and placed in sterile tubes containing 10 ml or 500 μl of distilled H_2_O, respectively. Lungs and spleen were homogenized for 30 s with a PowerGen 125 homogenizer (Fisher Scientific). Dilutions of the lung lysate and spleen lysate were plated on charcoal yeast extract agar for determination of colony-forming units per organ.

### Treatment of mice

In some experiments, mice were treated intranasally with 1 or 3 μg recombinant murine IL-33 (BioLegend) in a volume of 40 μl per mouse or PBS for 4 consecutive days (once a day). On day 6 after first IL-33 treatment, mice were inoculation with a sub-lethal dose of *L. pneumophila*. Mice were killed on day 2-post infection, and bacterial loads analysed. FACS analysis was performed on day 7-post the first inoculation of IL-33 to identify M2 macrophage populations. Cytokine analysis was performed on day 8 after IL-33 administration^17^. In another set of experiment, mice were treated with recombinant murine sST2, which was produced at the VIB Protein Service Facility, VIB Inflammation Research Center (Ghent, Belgium) and kindly provided by Dr Rudi Beyaert. Mice were injected subcutaneously with 100 μg of sST2 in a volume of 200 μl per mouse or PBS on days 1.5, 3, 5, 7, 9, 11 and 13 after CLP (once a day). At fifteenth day after CLP, surviving mice were challenged intranasally with a sublethal dose of *L. pneumophila*.

### Macrophage differentiation and polarization

BMDM were differentiated as described previously^17^. Bone marrow cells from WT mice were cultured in the presence of M-CSF (20 ng ml^−1^, R&D Systems) in a petri dish (BD OPTILUX). After 7 days, the macrophages (1 × 10^7^ per 10 ml) were stimulated with IL-4 (30 ng ml^−1^, R&D Systems), IL-13 (30 ng ml^−1^, R&D Systems), IL-33 (30 ng ml^−1^, BioLegend) (M2 differentiation); or LPS (1 μg ml^−1^, *E. coli* 0111:B4, Sigma-Aldrich), IFN-γ (200 ng ml^−1^, R&D Systems), M-CSF (5 ng ml^−1^, R&D Systems) (M1 differentiation); or M-CSF (5 ng ml^−1^, R&D Systems) alone (M0 macrophages) for 2 days. In some experiments, macrophages were cultured with 10 ng ml^−1^ IL-4 (R&D Systems) and/or 20 ng ml^−1^ IL-33 (BioLegend) for 2 days. At the end of cultures, the cells were used for FACS and supernatants for cytokine analysis by ELISA.

### M2 macrophages adoptive transfer

M0 or M2-polarized macrophages derived from bone marrow (BMDM) or vehicle (PBS) were injected i.v. to naive and sepsis-surviving WT and *Stat6*^*−/−*^ mice (4 × 10^6^ cells per mouse, on day 3 after CLP). The recipient mice were then infected i.n. with *L. pneumophila* on day 15 after CLP.

### Coculture of macrophages with T cells

Subsets of macrophages (M0, M1 or M2, 5 × 10^5^ per well) were co-cultured for 4 days with effector CD4^+^ T cells (CD4^+^ CD25^−^T cells, 5 × 10^5^ per well) purified from the spleen of WT or *Il10*^*−/−*^ naive mice, in the presence of IL-2 (10 ng ml^−1^, R&D Systems), anti-IL-10 (50 μg ml^−1^, clone JES052A5, R&D Systems) and stimulated with polyclonal anti-CD3 (1 μg ml^−1^, BD Biosciences) in U-bottom 96-well plates. Cells were then stained for Foxp3 and CD4 and analysed by FACS.

### Isolation of CD11b^+^ macrophage-enriched lung population

Lung tissue was digested with DNAse I (Sigma) and Liberase TL (Roche) for 45 min at 37 °C under rotation. Total lung cells were stained with specific antibody to anti-CD11b (M1/70, BioLegend) for 15 min. After that, cells were purified using anti-PE magnetic beads in AutoMACS (Miltenyi Biotec) according to manufacturer's instructions.

### ELISA

Cytokine concentrations in serum, lung and peritoneal lavages, and chemokine concentration in lung and culture supernatants were determined by ELISA according to each manufacturer's instructions. Antibody pairs or kits were obtained for IL-4, IL-10, IL-13 (eBioscience), IL-6 (BD Biosciences) and IL-1β, IL-33, IGF-1, TNF, CCL17, CCL22, Endocan-1 (R&D Systems). It was not specified in the manufacturer's datasheet if the IL-33 kit can distinguish between pro-IL-33 and cleaved IL-33.

### Western blotting

Arginase 1 expression in peritoneal cells was determined by western blotting according to manufacturer's directions. Briefly, the nitrocellulose membrane was incubated with polyclonal sheep anti-mouse Arginase 1 (#AF5868, R&D Systems) at 4 °C overnight, followed by incubation with anti-sheep IgG, horseradish peroxidase-linked secondary antibody and developed using enhanced chemiluminescence technique (ChemiDoc XRS System, Bio-Rad Laboratories). The membrane was stripped and reprobed with Gapdh as a loading control. Full scan of the original uncropped western blot is shown in Supplementary Fig. 13.

### Gene expression by qPCR

Total RNA from lung tissue was extracted using the TRIZOL reagent (Invitrogen) according to manufacturer's directions. For the CD11b^+^ cells isolated from lung tissues, the total RNA was extracted using RNeasy Mini Kit (Qiagen) according to manufacturer's instructions. Total RNA (2 μg for lung tissue and 400 ng for CD11b^+^ cells) was reverse-transcribed using high capacity cDNA RT Kit (Thermo Fisher Scientific). Quantitative real-time PCR was performed using TaqMan Gene Expression Master Mix or Power Syber Green PCR Master Mix TaqMan (Thermo Fisher Scientific) and the Viia7 Real-Time PCR system. All data were normalized to Gapdh values; and results were analysed using comparative Ct method (or ΔΔCt method). The relative gene expression is presented as the fold-increase over that seen in naive WT controls. qPCR was performed using Taqman primers (Thermo Fisher Scientific) for *Cebpb* (Mm00843434_s1), *Arg1* (Mm00475988_m1), *Mrc1* (Mm00485148_m1), *Igf1* (Mm00439560_m1) and *Gapdh* (Mm99999915_g1). For the CD11b^+^ cells isolated from lung tissues, qPCR was performed using syber primers for *Retnla* (Foward: CCT GAG ATT CTG CCC CAG GAT, reverse: TTC ACT GGG ACC ATC AGC TGG), *Mrc1* (Forward: CTC GTG GAT CTC CGT GAC AC, reverse: GCA AAT GGA GCC GTC TGT GC) and Gapdh (forward: GGG TGT GAA CCA CGA GAA AT, reverse: CCT TCC ACA ATG CCA AAG TT).

### Flow cytometry

Aliquots of freshly harvested cells (1 × 10^6^ cells per tube) were suspended in buffer containing 2% FCS in PBS. To stain for surface antigens, cells were incubated with specific antibodies to F4/80 (BM8, eBioscience), CD206 (mannose receptor C type 1, MR; MR5D3, AbD Serotec), CD4 (GK1.5, eBioscience; H129.19, BD Biosciences), CD4 (RPA-T4, BD Biosciences, for human), CD45 (30-F11, BD Biosciences), Ly-6A/E (Sca-1; E13-161.7, BioLegend), CD3e (145-2C11, BD Biosciences), CD8a (53-6.7, eBioscience), CD19 (eBIO1D3, 1D3, eBioscience; 6D5, BioLegend), CD11b (M1/70, BioLegend), CD11c (N418, BioLegend; Hl3, BD Biosciences), CD49b/Pan-NK (DX5, BD Biosciences), FcɛRI Alpha (MAR-1, eBioscience), T1/St2 (IL-33R, DJ8, MD Biosciences) or the appropriate isotype controls for 30 min. To stain for intracellular murine antigens, cells were first stained for surface antigens, then fixed and permeabilized with mouse Foxp3 Buffer Set (BD Biosciences), according to the manufacturer's recommendations. Cells were then incubated with specific antibodies to FoxP3 (FJK-16 s, eBioscience), FoxP3 (150D/E4, eBioscience, for human) or IL-13 (eBio13A, eBioscience) for 45 min. For intracellular IL-13 staining, cells were incubated with phorbol-12-myristate-13-acetate (Sigma-Aldrich), ionomycin (750 ng ml^−1^, Sigma-Aldrich) and GolgiStop (BD Biosciences) for 4 h. To stain pSTAT6, cells were incubated with rmIL-4 (30 ng ml^−1^, R&D Systems) or medium for 15 min at 37 °C. Cells were then washed in PBS, fixed immediately in formaldehyde (final concentration 2%) for 10 min at 37 °C, washed and re-suspended in ice-cold methanol for 30 min at 4 °C. Cells were washed twice in staining buffer and stained with an antibody specific for pSTAT6 (pY641, BD Biosciences) and F4/80 (BM8, eBioscience) or the appropriate isotype controls. Cells were analysed by FACSCanto using FCS Express V3 (De Novo Software, Los Angeles, CA). Gating strategies for flow cytometry analysis are shown in Supplementary Fig. 14.

### Intracellular growth of bacteria on macrophage

Macrophages from bone marrow–derived macrophages (BMDM) or peritoneal cells were added to 24-well plates at a density of 2.5 × 10^5^ cells per well. *L. pneumophila* bacteria were added to each well at an MOI of 0.1 in the presence of BEC (S)-(2-Boronoethyl)-l-cysteine) or medium alone. On the third day, macrophages were lysed in sterile H_2_O, and the cell lysates were plated for counted of bacteria colonies present after 4 days of incubation at 37 °C (ref. 57). The results are expressed as log of colony-forming units (log CFU per ml).

### Measurements of organ damage biomarkers

Liver injury was assessed by measuring the increase in serum concentrations of alanine aminotransferase; renal dysfunction was assessed by the increase in blood urea nitrogen, and creatine kinase-MB was used as an index of cardiac lesions. The determinations were performed using a commercial kit (Labtest).

### Statistical analysis

Prism 5 software (GraphPad Software) was used to analyse data by two-tailed unpaired Student's *t*-tests. When CLP groups were compared with a control group, one-way ANOVA result with Dunnett posthoc tests was performed. Data are presented as means±s.e.m. Survival studies were analysed with the Mantel–Cox log-rank test, and bacterial counts were analysed by the Mann–Whitney *U* test and presented as median. *P<*0.05 was considered statistically significant. No pre-specified effect size was assumed, and in general three mice or replicates for each group were used in experiments; this sample size was sufficient to demonstrate statistically significant differences in comparisons between two unpaired experimental groups by an unpaired *t*-test, multiple comparations by one-way ANOVA result with Bonferroni posthoc tests or comparison with a control group, one-way ANOVA result with Dunnett posthoc tests. Since all mouse strains were generated and maintained on the same original inbred background (BALB/c or C57BL/6 J), the variation within each data set obtained by experiments with primary cells or mice was assumed to be similar between genotypes. However, experimental assessment of variance was not performed. Mice were to be excluded from analysis only if they displayed obvious illness or death. No randomization was used in these studies. Human experiments were blinded on samples group. However, we were not blinded to genotypes or group allocations during animal experiments.

### Data availability

The data supporting the findings of this study are available within the article and its supplementary information files or from the corresponding authors on reasonable request.

## Additional information

**How to cite this article:** Nascimento, D. C *et al*. IL-33 contributes to sepsis-induced long-term immunosuppression by expanding the regulatory T cell population. *Nat. Commun.* **8**, 14919 doi: 10.1038/ncomms14919 (2017).

**Publisher's note:** Springer Nature remains neutral with regard to jurisdictional claims in published maps and institutional affiliations.

## Supplementary Material

Supplementary Information Supplementary Figures and Supplementary Tables
