# Supplementary material for: IL-33 contributes to sepsis-induced long-term immunosuppression by expanding the regulatory T cell population
Source: Nat Commun. 2017 Apr 4;8:14919. doi: 10.1038/ncomms14919 (PMC5382289; doi:10.1038/ncomms14919)
Supplement: Supplementary Information — Supplementary Figures and Supplementary Tables [file ncomms14919-s1.pdf]

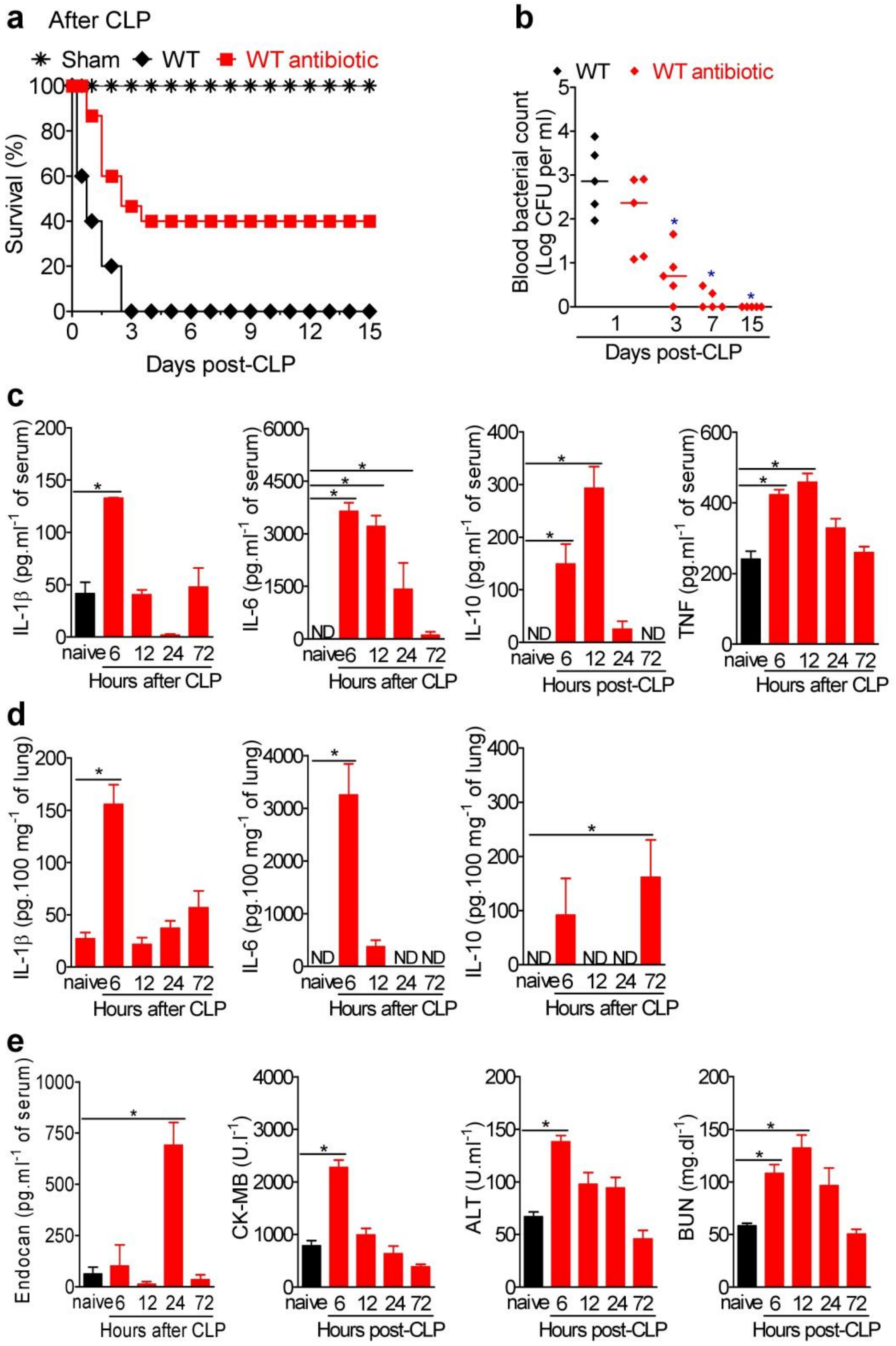

a
After CLP
Sham
WT
WT antibiotic
Survival (%)
Days post-CLP
b
WT
WT antibiotic
Blood bacterial count (Log CFU per ml)
Days post-CLP
c
IL-1β (pg.ml-1 of serum)
IL-6 (pg.ml-1 of serum)
IL-10 (pg.ml-1 of serum)
TNF (pg.ml-1 of serum)
Hours after CLP
Hours post-CLP
d
IL-1β (pg.100 mg-1 of lung)
IL-6 (pg.100 mg-1 of lung)
IL-10 (pg.100 mg-1 of lung)
Hours after CLP
e
Endocan (pg.ml-1 of serum)
CK-MB (U.l-1)
ALT (U.ml-1)
BUN (mg.dl-1)
Hours after CLP
Hours post-CLP

Supplementary Figure 1. Antibiotic partially rescues mice from sepsis. (**a-b**) BALB/c mice under CLP were treated with antibiotic or PBS. (**a**) Survival curves. WT Sham (n=5), WT CLP or WT CLP antibiotic (n=20). (**b**) Bacterial load in the blood after CLP. (**c-d**) Serum and lungs of C57BL/6J mice under CLP were collected at the indicated times points. IL-1β, IL-6 and IL-10 concentrations in serum (**c**) and lung tissue (**d**) were determined by ELISA.(**e**) Serum endocan concentration was determined by ELISA and the levels of creatine kinase-MB (CK-MB), alanine aminotransferase (ALT) and blood urea nitrogen (BUN) were determined by colorimetric assays. ND, not detected. *$p < 0.05$ compare with naïve control or day 0 (**b**) (Mantel-Cox log-rank test in **a**, Mann-Whitney U test in **b** and one-way ANOVA result with Dunnett posthoc tests in **c-e**). Data are representative of three (**a-b**) or two (**a, b, g**) (mean ± s.e.m. in **c-e** and median in **b**).

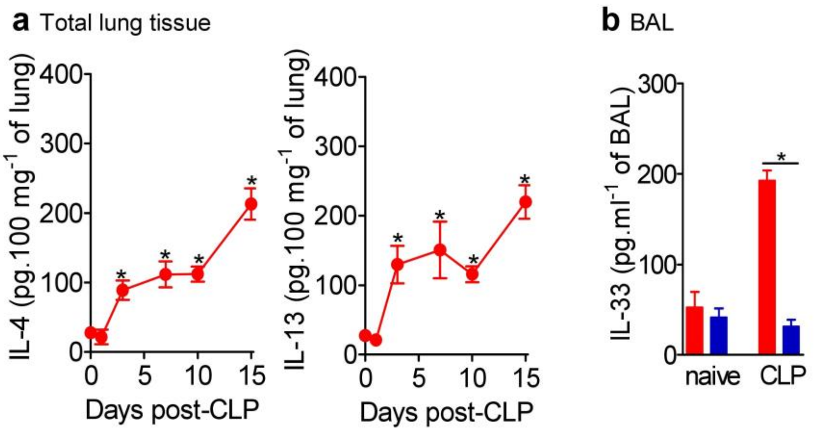

Supplementary Figure 2. Kinetics of type-2 cytokines production during CLP. Lungs and bronchial lavage (BAL) fluids of mice under CLP and treated with antibiotic were collected at the indicated times points. (**a**) IL-4 and IL-13 concentrations in the lung tissues of C57BL/6J were determined by ELISA. (**b**) IL-33 concentrations in the BAL of BALB/c and *Il1rl1*$^{-/-}$ at day 15 after CLP were determined by ELISA. *p < 0.05 and **p < 0.01, comparing groups as indicated or with day 0 (one-way ANOVA result with Dunnett posthoc tests in **a** and two-tailed unpaired Student's *t*-test in **b**). Data are mean ± sem of 3-10 mice/group.

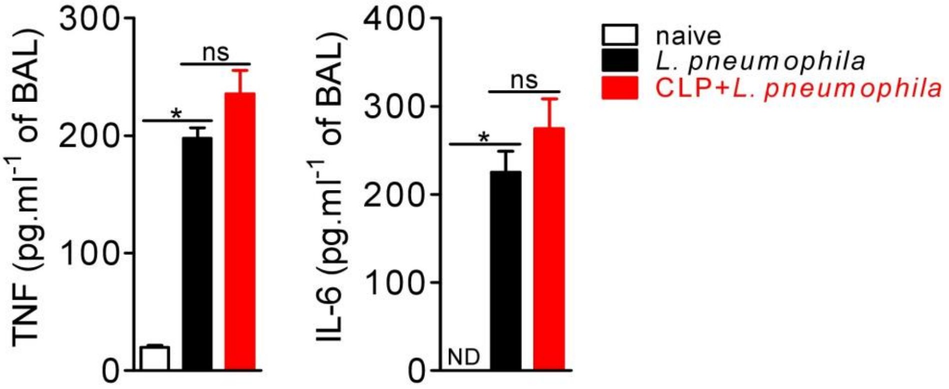

Supplementary Figure 3. Proinflammatory cytokines production during secondary infection in sepsis-surviving mice. C57BL/6J mice were subjected to CLP and challenged i.n. 15 days later with *L. pneumophila.* TNF and IL-6 concentrations in the BAL were determined by ELISA 12 h after challenge. ND, not detected. *$p < 0.05$ (two-tailed unpaired Student’s *t*-test), ns, not significant. Data are mean ± sem of 3-5 mice/group.

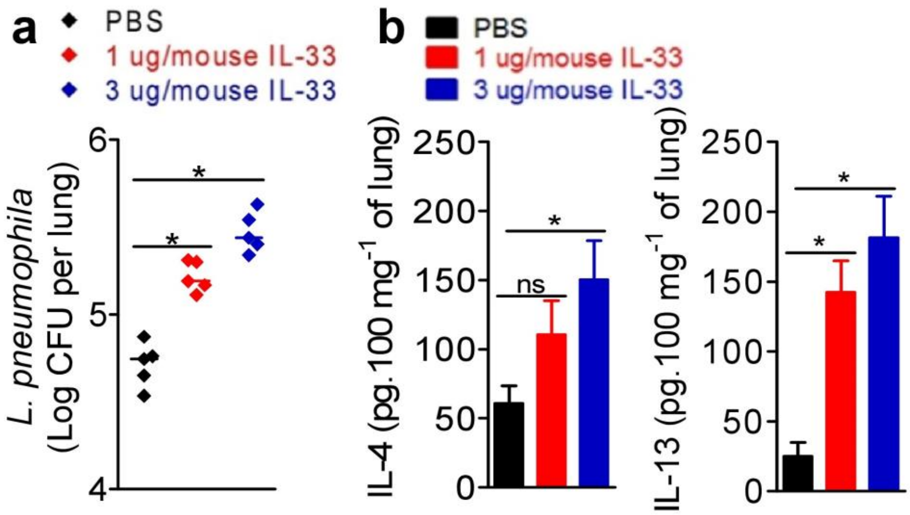

Supplementary Figure 4. Repeated administration of IL-33 impaired bacterial clearance and increased type-2 cytokines production. BALB/c mice were inoculated i.n. with 1 or 3 µg of IL-33 or PBS for 4 consecutive days and challenged i.n. 2 days later with *L. pneumophila*. (**a**) Bacterial load in the lung tissue 48 h after challenge (*n* = 5 mice per group). (**b**) IL-4 and IL-13 concentrations in the lung tissue 48 h after challenge (*n* = 5 mice per group). *p < 0.05 (Mann-Whitney U test in **a** and two-tailed unpaired Student's *t*-test in **b**), ns, not significant. Data are median (**a**) and mean ± sem (**b**) of 5 mice/group.

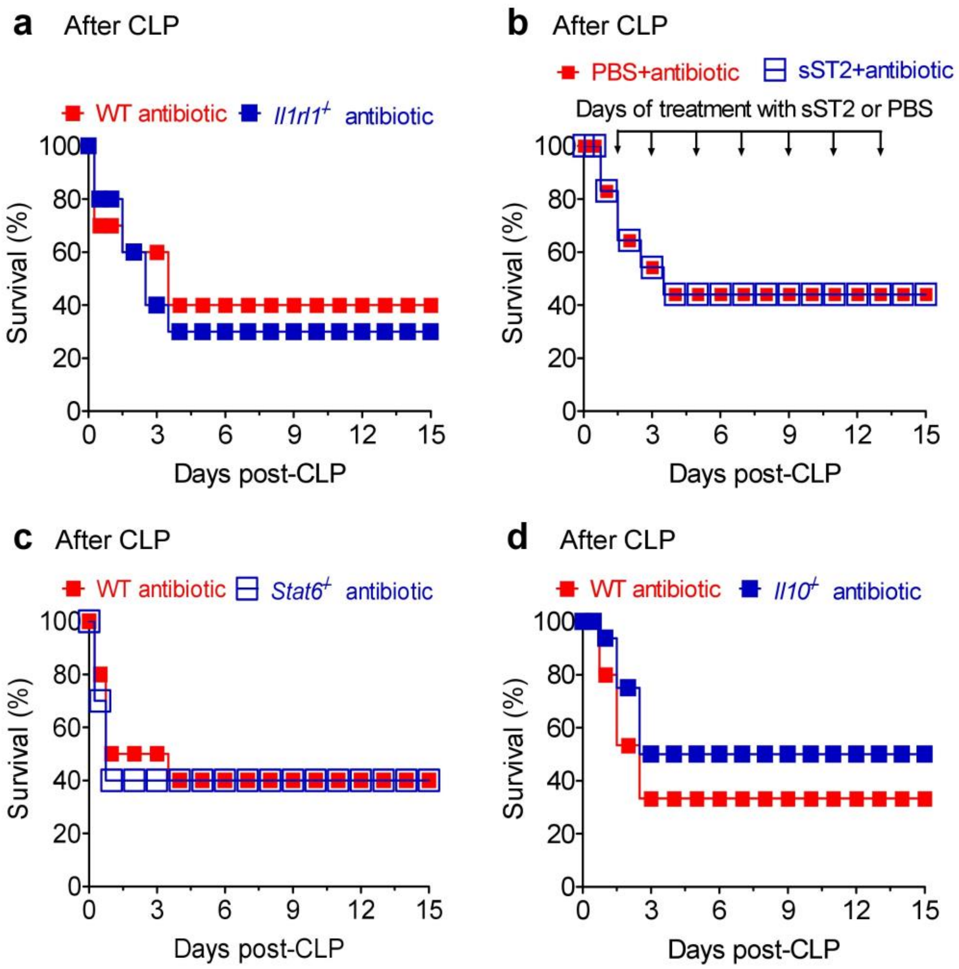

Supplementary Figure 5. WT, *Il1rl1*$^{-/-}$, *Stat6*$^{-/-}$ and *Il10*$^{-/-}$ mice show similar survival curves following CLP and antibiotic treatment. Survival curves after CLP of (**a**) BALB/c and *Il1rl1*$^{-/-}$ mice (*n* = 40 for WT group and *n* = 45 mice for *Il1rl1*$^{-/-}$ CLP group), (**b**) BALB/c mice treated with sST2 or PBS (*n* = 15 mice per group), (**c**) BALB/c and *Stat6*$^{-/-}$ mice (*n* = 40 for WT group and *n* = 42 mice for *Stat6*$^{-/-}$ CLP group), and (**d**) C57BL/6J *and Il10*$^{-/-}$ mice (*n* = 40 for WT group and *n* = 36 mice for *Il10*$^{-/-}$ CLP group). Data are percentage of survival from 1 experiment (**b**) or pooled from three (**d**), five (**c**) or six (**a**) experiments.

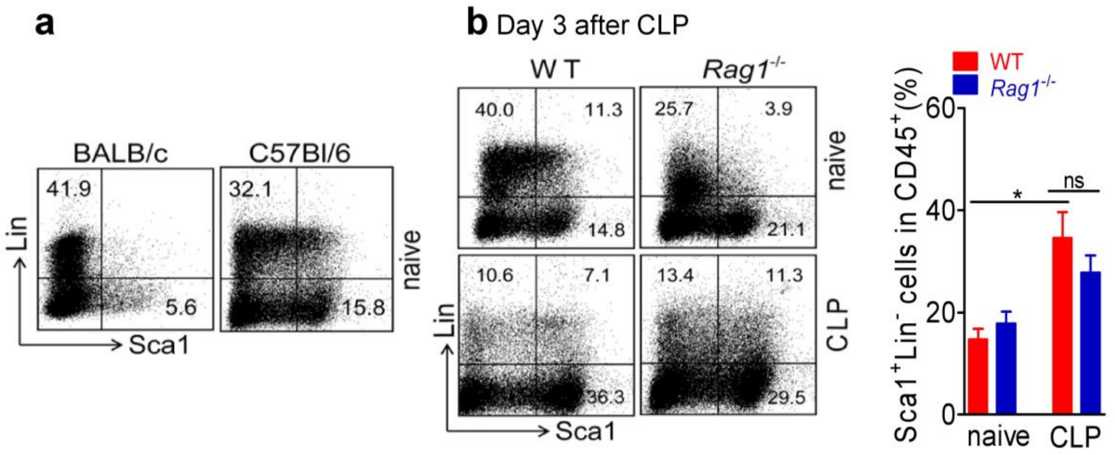

Supplementary Figure 6. Induction of ILC2 in *Rag1*$^{-/-}$ sepsis-surviving mice. Lungs of C57BL/6J and *Rag1*$^{-/-}$ mice under CLP and antibiotic treatment were collected on day 3 after CLP. (**a**) Representative FACS plots of ILC2^{+} cells (Sca1^{+} in Lin^{-}CD45^{+} cells) from BALB/c and C57BL/6J mice was determined by FACS ($n \geq 3$ mice per group). Representative FACS plots from one experiment. (**b**) Representative FACS plots and frequency of ILC2^{+} cells (Sca1^{+} in Lin^{-}CD45^{+} cells) from C57BL/6J and *Rag1*$^{-/-}$ mice was determined by FACS ($n \geq 3$ mice per group). *$p < 0.05$ (two-tailed unpaired Student's *t*-test in **b**), ns, not significant. Data are mean ± sem of 3-5 mice/group.

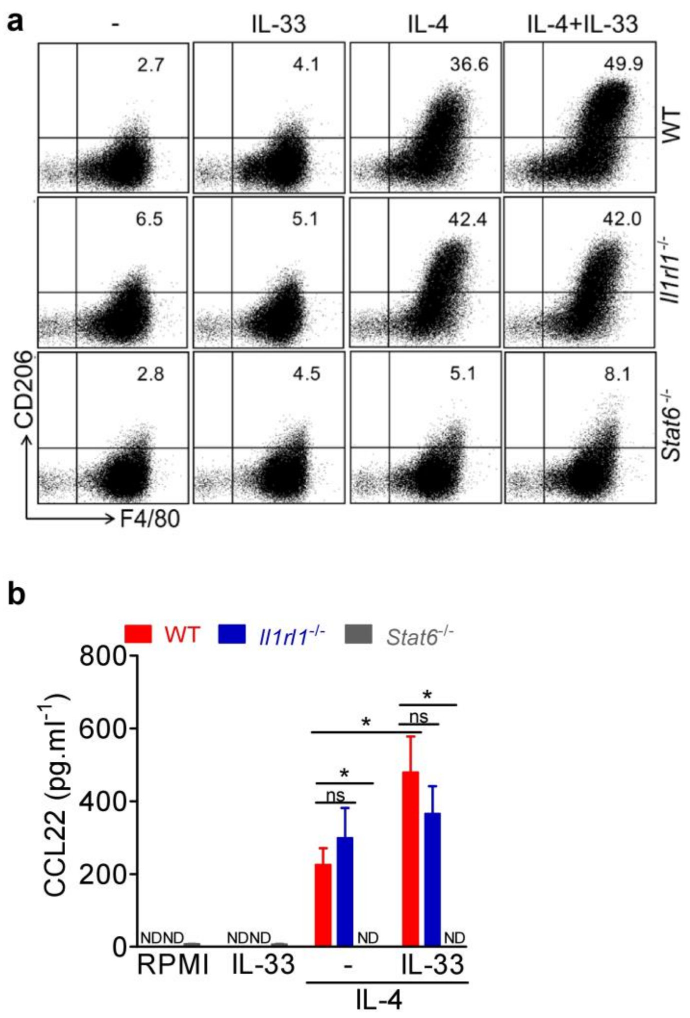

Supplementary Figure 7. M2 polarization with IL-4 ± IL-33 was abolished in *Stat6*$^{-/-}$ BMDM *in vitro*. BMDM from BALB/c, BALB/c *Stat6*$^{-/-}$ *or* BALB/c *Il1rl1*$^{-/-}$ mice were cultured for 2 days in the presence of IL-4 and/or IL-33, or medium alone. (**a**) Percentage of F4/80^{+}CD206^{+} cells was analysed by FACS (each cell type was pooled from 2 mice and from 2 well per group). (**b**) Concentrations of CCL22 in the 2 days culture supernatants determined by ELISA (each cell type was pooled from 2 mice and 3-5 well per group). ND, not detected. *$p < 0.05$ (two-tailed unpaired Student's *t*-test in **b**), ns, not significant.

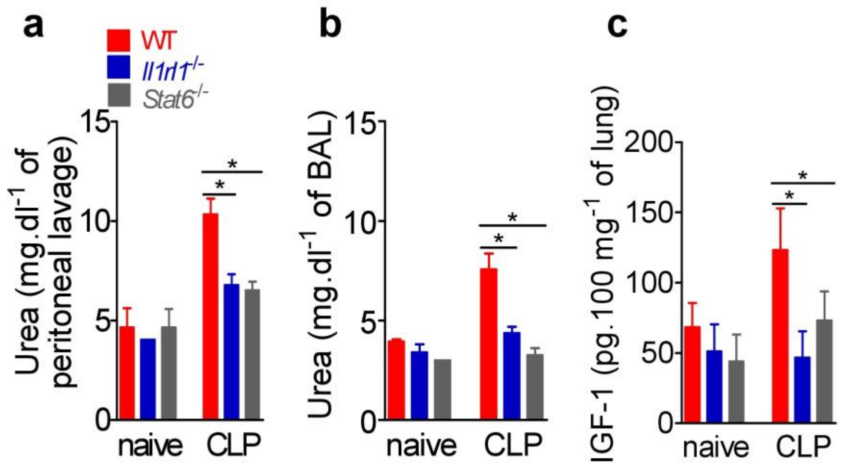

Supplementary Figure 8. Production of urea, IGF-1 and TGF-β of macrophages from sepsis-surviving mice. Peritoneal lavage fluid and lungs from BALB/c, *Il1rl1*$^{-/-}$ and *Stat6*$^{-/-}$ mice under CLP and antibiotic treatment were collected on day 15 after CLP. (**a-b**) Urea concentrations in the peritoneal lavage (**a**) and BAL (**b**) were determined by colorimetric assays. (**c**) IGF-1 concentrations in the lung tissue were determined by ELISA. *$p < 0.05$ and **$p < 0.01$ (two-tailed unpaired Student's *t*-test). Data are representative of two independent experiments (mean ± sem of 3–8 mice/group).

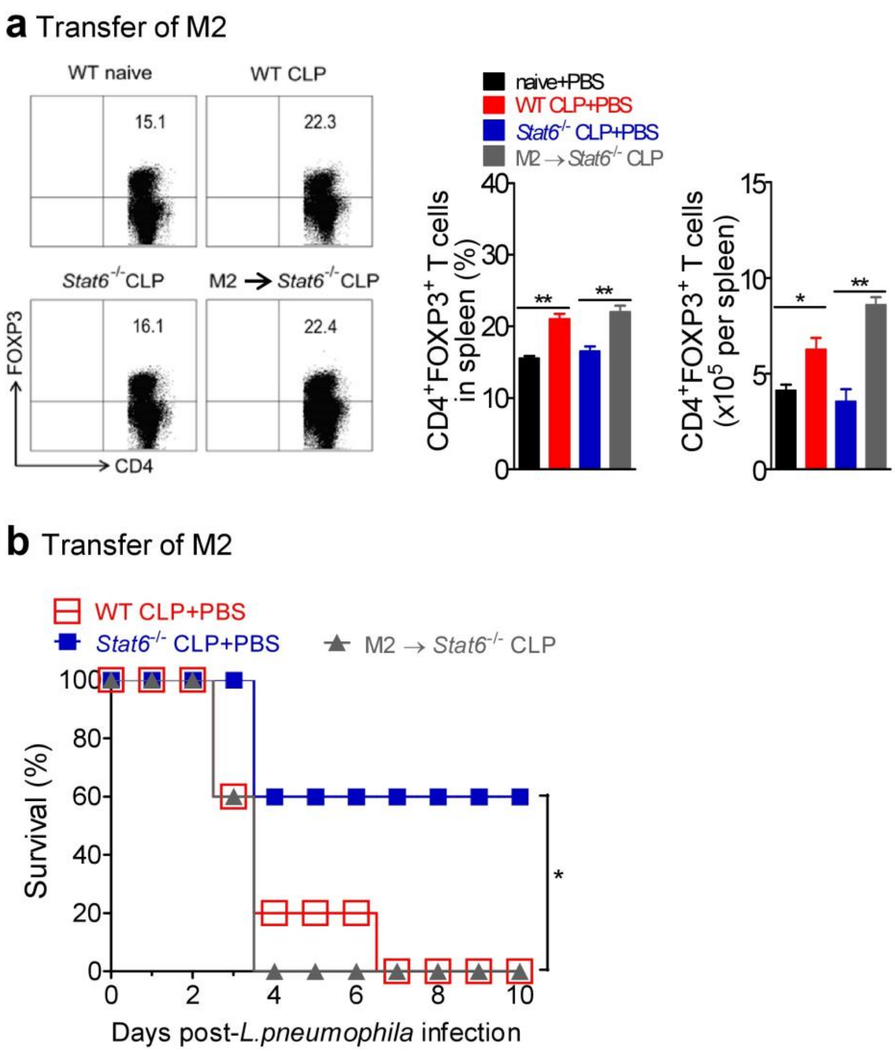

Supplementary Figure 9. M2 macrophage induces expansion of Treg cell population in sepsis-surviving mice. PBS or M2 macrophages were adoptively transferred ($4x10^6$ cells, i.v., on day 3 after CLP) into BALB/c or *Stat6*$^{-/-}$ sepsis-surviving mice. The surviving mice were either sacrificed or challenged with *L. pneumophila* on day 15 after CLP. (**a**) Representative FACS plots, frequency and number of Foxp3^{+} CD4^{+} T cells (*n* = 3 mice per group). (**b**) Survival curves of mice after *L. pneumophila* challenge (*n* = 10 mice per group). *p < 0.05 and **p < 0.01 (two-tailed unpaired Student's *t* test in **a**, Mantel-Cox log-rank test in **b**). Data are representative of two (**a**) independent experiments or are pooled of two (**b**) experiments (mean ± s.e.m. in **a**).

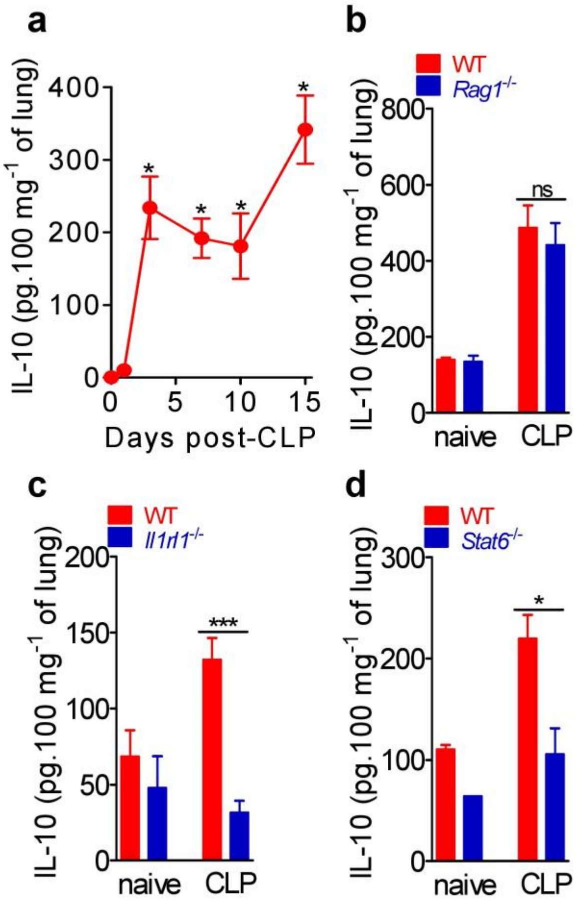

Supplementary Figure 10. IL-10 production in the lungs of sepsis-surviving mice. (**a**) Lung tissues from C57BL/6J mice under CLP and antibiotic treatment were collected at the indicated times points and IL-10 concentrations determined by ELISA.(**b-d**) Lung tissues of C57BL/6J, *Rag1*$^{-/-}$ (**b**), BALB/c, *Il1rl1*$^{-/-}$ and *Stat6*$^{-/-}$ (**c-d**) mice under CLP and antibiotic treatment were collected on day 15 after CLP and IL-10 concentrations determined by ELISA. *$p < 0.05$, ***$p < 0.001$ comparing groups as indicated or day 0 (**a**) (one-way ANOVA result with Dunnett posthoc tests in **a** and two-tailed unpaired Student's *t*-test in **b-d**). Data are representative of two independent experiments (mean ± sem of 3–10 mice/group).

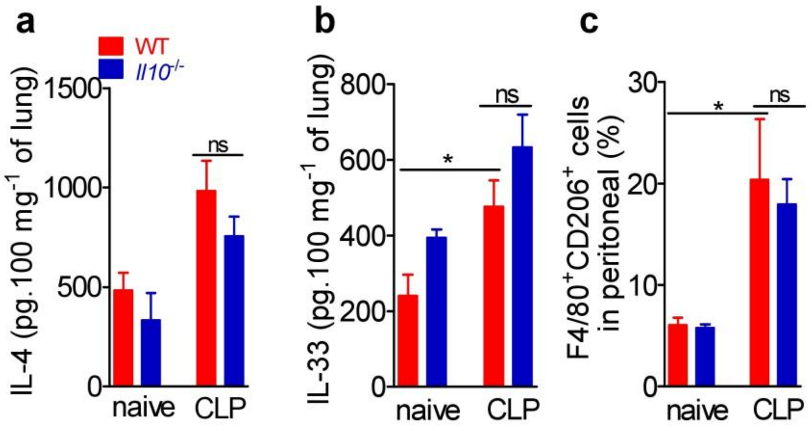

Supplementary Figure 11. C57BL/6J and *Il10*$^{-/-}$ mice produced similar levels of type-2 cytokines and frequency of M2 macrophages during sepsis. Lungs and peritoneal lavage fluids from C57BL/6J and *Il10*$^{-/-}$ mice under CLP and antibiotic treatment were collected on day 15 after CLP. IL-4 (**a**) and IL-33 (**b**) concentrations in the lung tissue were determined by ELISA. (**c**) Frequency of peritoneal F4/80^{+}CD206^{+} macrophages was determined by FACS. *$p < 0.05$ (two-tailed unpaired Student's *t*-test), ns, not significant. Data are representative of two independent experiments (mean ± sem of 3–12 mice/group).

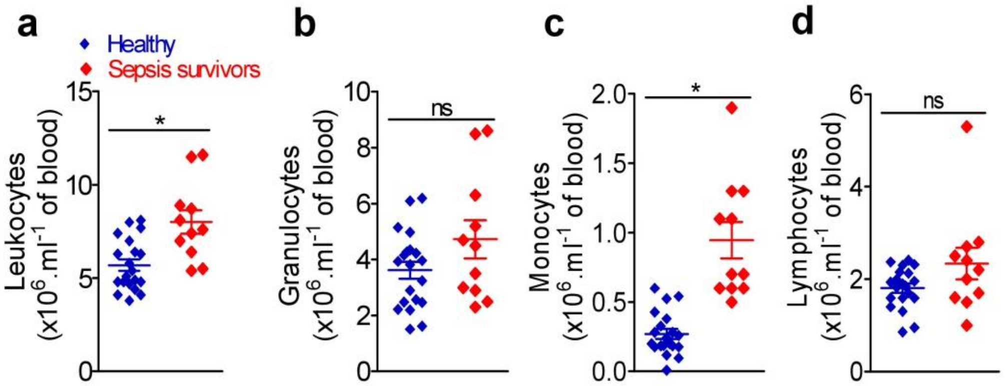

Supplementary Figure 12. Differential cell counts of the peripheral blood of septic patients. Peripheral blood from sepsis-surviving patients (n=11) and healthy controls (*n* = 14) was examined for (**a**) leukocytes, (**b**) granulocytes, (**c**) monocytes and (**d**) lymphocytes. Data are mean ± s.e.m. *p < 0.05 (two-tailed unpaired Student's *t*-test), ns, not significant.

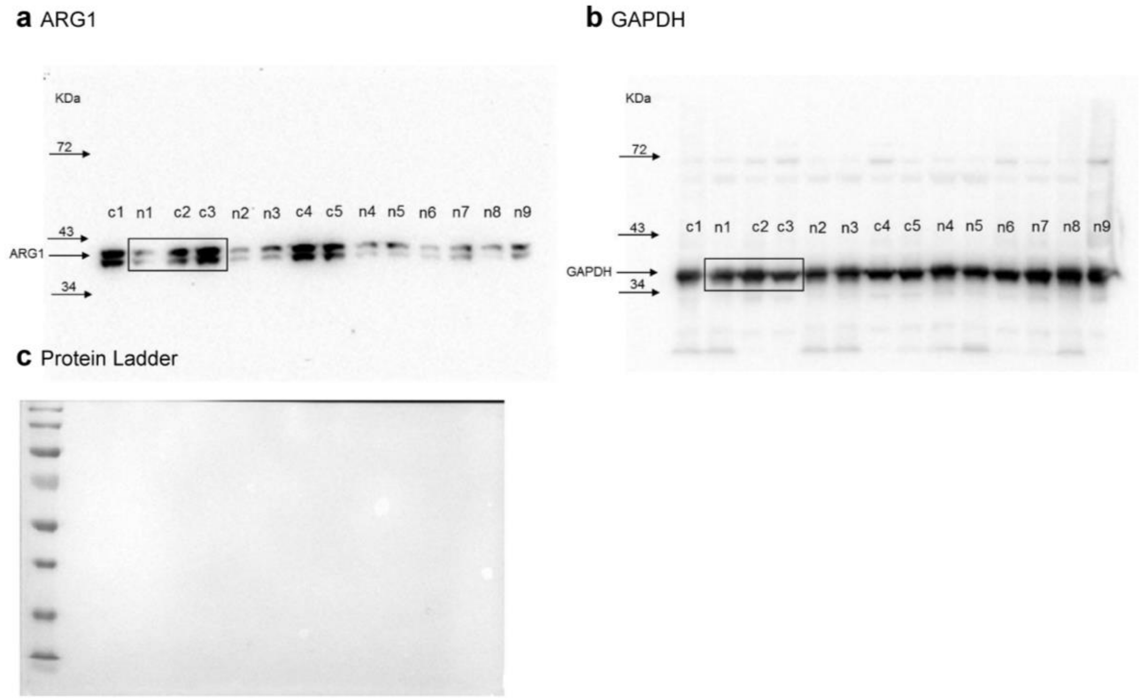

Supplementary Figure 13. Full scan of the original uncropped western blot. Original Western blots used in Fig. 4e. (**a**) Western blot for Arg1. (**b**) Western blot for GAPDH. (**c**) Protein Ladder. n, naïve mice (n=9 mice). c, CLP mice (n=5 mice).

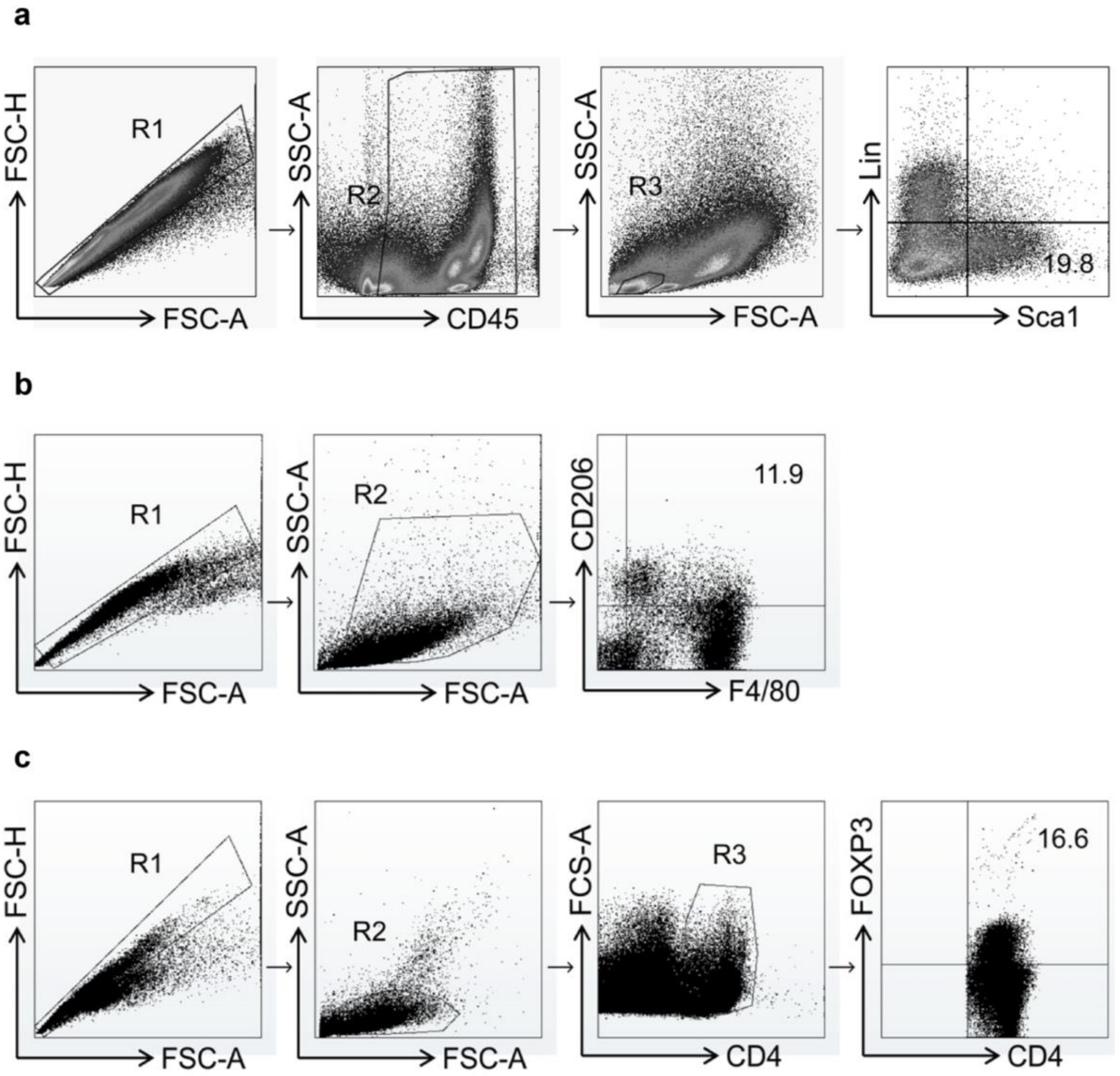

Supplementary Figure 14. Representative gating strategies used for flow cytometry analysis. Doublets were excluded by FSC-H and FSC-A gating for all flow cytometry analysis. (**a**) Proportion of ILC2 cells stained for ST2$^+$Lin$^-$ (CD3$^-$CD11c$^-$CD19$^-$F4/80$^-$) among CD45$^+$ cells in single cell suspensions of lung cells. (**b**) Proportion of M2 macrophages stained for CD206$^+$ and F4/80$^+$ in peritoneal cells. (**c**) Proportion of Treg cells stained for FOXP3$^+$ among CD4$^+$ cells in spleen cells.

Supplementary Table 1

Demographic and clinical characteristics of septic patients

**Patients**	**n=11**
Age (years) - mean (SD)	60.2 (±12.83)
Female/Male	4/7
APACHE II - mean (SD)	17.72 (±9.73)
SOFA - mean (SD)	9.81 (±4.87)
SAP3 - mean (SD)	45.7 (±23.98)

SD: standard deviation, APACHE II: Acute Physiology and Chronic Health Evaluation II, SOFA: Sepsis-related Organ Failure Assessment, SAP3: Simplified Acute Physiology score 3.

Supplementary Table 2

Clinical characteristics of septic patients

Patients	Site of Infection	Time after sepsis (months)	Severity of sepsis	Microorganisms isolated
**1**	Respiratory	5	Septic Shock	*Staphylococcus haemolyticus*
2	Respiratory	7	Severe Sepsis	*Enterococcus faecium*
3	Respiratory	7	Severe Sepsis	*Escherichia coli*
4	Respiratory	10	Septic Shock	*Streptococcus pneumoniae*
5	Respiratory	9	Severe Sepsis	Negative
6	Respiratory	7	Septic Shock	Negative
7	Respiratory	6	Severe Sepsis	Negative
8	Respiratory	5	Septic Shock	*Staphylococcus haemolyticus*
9	Skin	5	Septic Shock	Negative
10	Urinary	9	Severe Sepsis	*Staphylococcus aureus*
11	Respiratory	7	Severe Sepsis	Negative
